# The nature of activatory and tolerogenic dendritic cell-derived signal II

**DOI:** 10.3389/fimmu.2013.00053

**Published:** 2013-02-28

**Authors:** Ghaith Bakdash, Simone P. Sittig, Tjeerd van Dijk, Carl G. Figdor, I. Jolanda M. de Vries

**Affiliations:** Department of Tumor Immunology, Nijmegen Centre for Molecular Life Sciences, Radboud University Nijmegen Medical CentreNijmegen, Netherlands

**Keywords:** activation, tolerance, co-stimulation, co-inhibition, dendritic cells, T cell priming

## Abstract

Dendritic cells (DCs) are central in maintaining the intricate balance between immunity and tolerance by orchestrating adaptive immune responses. Being the most potent antigen presenting cells, DCs are capable of educating naïve T cells into a wide variety of effector cells ranging from immunogenic CD4^+^ T helper cells and cytotoxic CD8^+^ T cells to tolerogenic regulatory T cells. This education is based on three fundamental signals. Signal I, which is mediated by antigen/major histocompatibility complexes binding to antigen-specific T cell receptors, guarantees antigen specificity. The co-stimulatory signal II, mediated by B7 family molecules, is crucial for the expansion of the antigen-specific T cells. The final step is T cell polarization by signal III, which is conveyed by DC-derived cytokines and determines the effector functions of the emerging T cell. Although co-stimulation is widely recognized to result from the engagement of T cell-derived CD28 with DC-expressed B7 molecules (CD80/CD86), other co-stimulatory pathways have been identified. These pathways can be divided into two groups based on their impact on primed T cells. Whereas pathways delivering activatory signals to T cells are termed co-stimulatory pathways, pathways delivering tolerogenic signals to T cells are termed co-inhibitory pathways. In this review, we discuss how the nature of DC-derived signal II determines the quality of ensuing T cell responses and eventually promoting either immunity or tolerance. A thorough understanding of this process is instrumental in determining the underlying mechanism of disorders demonstrating distorted immunity/tolerance balance, and would help innovating new therapeutic approaches for such disorders.

## INTRODUCTION

The immune system is endowed with the unique capacity to protect against invading pathogens, yet not react to self. Among the different constituents of the immune system, dendritic cells (DCs) play a central role in drawing the thin line between immunity and tolerance. Discovered in 1973 (Steinman and Cohn, 1973), DCs are recognized as the most potent antigen presenting cells (APCs). Their ability to initiate and modulate various forms of T cell responses, earned them the position of being master orchestrators of adaptive immunity (Banchereau and Steinman, 1998). DCs are spread throughout the body, residing in different tissues as sentinels, monitoring their surrounding environment for any signs of danger. Equipped with pathogen recognition receptors (PRRs), DCs are capable of sensing pathogenic invasion (Medzhitov and Janeway Jr., 2002) and self-structures associated with cellular stress (Matzinger, 2002). Upon danger sensing, DCs will undergo functional changes, also known as maturation, crucial for the ensuing induction of T cell responses (Banchereau et al., 2000). A hallmark of DC maturation is the expression of the chemokine receptor CCR7 that allows mature DCs to migrate to draining lymphoid tissues where they activate naïve T cells in a process based on three signals. The first signal results from the ligation of T cell receptors (TCRs) to pathogen-derived peptide antigens that are presented by major histocompatibility complex (MHC) molecules of DCs, which are upregulated upon maturation. This principal stimulation signal is important to assure antigen specificity of the immune response. Although TCR triggering is crucial for naïve T cell activation, it is not sufficient by itself to initiate an efficacious immune response. The concept of a second co-stimulatory signal was first introduced by Lafferty and Woolnough (1977). They deduced from organ transplantation studies that alloantigens presented by transplanted tissues failed to elicit any immune responses unless accompanied by hematopoietic stimulator cells (Lafferty and Woolnough, 1977). This concept was corroborated by seminal observations by the group of Schwartz, implying that T cells activated solely by TCR engagement were rendered unresponsive and anergic (Jenkins and Schwartz, 1987). This was followed by the discovery of the main elements of co-stimulation: CD28 (Aruffo and Seed, 1987) and CD80 (Freeman et al., 1989), the latter being initially identified as a B cell activation marker and eventually recognized as the ligand of CD28 (Linsley et al., 1990). Subsequently, more pathways contributing to signal II were identified. Based on the nature of their signal, these molecules can be divided into co-stimulatory molecules that promote T cell proliferation, and co-inhibitory molecules that attenuate T cell responses. The nature of signal II is vital in determining the T cell response, which is further defined by a third polarizing signal. This third signal promotes the selective development of naïve T cells into one of the identified types of effector or tolerogenic T cells (De Jong et al., 2005). Although signal III is generally recognized to be mediated by soluble DC-derived cytokines, there are indications that signal II may also contribute to T cell polarization. A final putative DC-derived signal is suggested to provide polarized T cells with homing directions to the site of infection or injury (Sigmundsdottir and Butcher, 2008). Thus, DCs control the delicate balance between immunity and tolerance through the signals they convey to T cells.

**FIGURE 1:**
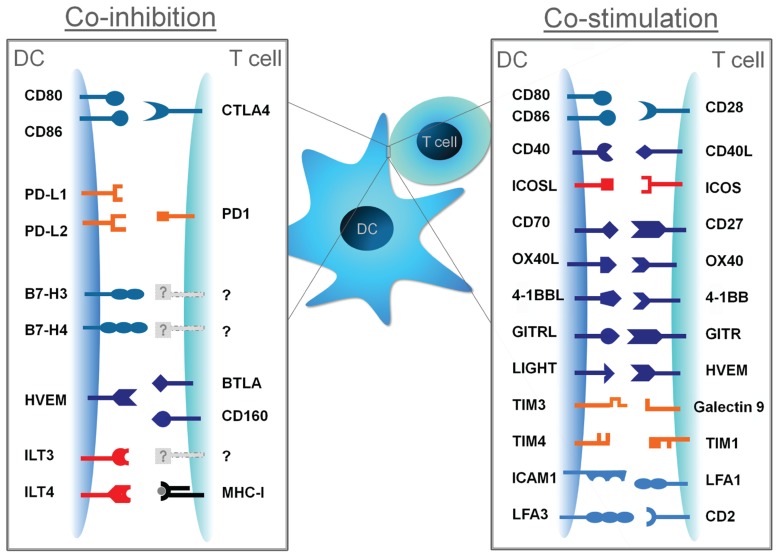
**Co-stimulatory and co-inhibitory molecules and their cognate ligands**. DC-derived signal II can promote T cell activation when conveyed by co-stimulatory molecules, or can attenuate T cell responses when conveyed by co-inhibitory molecules.

Although the combined effect of all DC-derived signals is important for full blown T cell responses, signal II is key for allowing these responses and licensing them to become either immunogenic or tolerogenic. Here, we shed light on the multifaceted signal II by reviewing current knowledge of to date identified co-stimulatory and co-inhibitory pathways (**Figure 1**), their mode of action, relation to disease, and any possible clinical applications based on utilizing these pathways.

## CO-STIMULATORY MOLECULES

### CD80/CD86/CD28 PATHWAY

Following the discovery of the CD80/CD28 interaction, B7-2 (CD86) was identified as a second ligand for CD28 (Azuma et al., 1993). The CD80/CD86/CD28 pathway was suggested to deliver the strongest co-stimulatory pathway as CD28-deficient cells failed to proliferate in the presence of APCs (Green et al., 1994). The consequences of CD28 engagement by its ligands comprise stimulation of T cell proliferation, dramatic upregulation of IL-2 (Linsley et al., 1991a), promotion of T cell survival by enhancing Bcl-XL expression (Boise et al., 1995), and enhanced glycolytic flux to meet energetic requirements associated with a sustained response (Frauwirth et al., 2002). Those effects were shown to be dependent on activating the signaling cascades of phosphoinositide-3 kinase (PI3K), protein kinase B (PKB, also known as Akt), and nuclear factor kappaB (NF-κB; Song et al., 2008).

Several reports pointed out a possible role for CD28 signaling in T cell polarization. Murine T cells were shown to produce enhanced levels of IL-4 and IL-5, characteristic for T helper (Th) 2, upon strong CD28 stimulation (Rulifson et al., 1997). Strong CD28 signaling was also demonstrated to inhibit Th17 responses (Purvis et al., 2010). Although it is generally accepted that memory T cells, unlike naives, are less dependent on co-stimulation via CD28, it was shown that this co-stimulatory pathway is important in controlling T cell recall responses (Ndejembi et al., 2006).

In addition to its key role in initiating and sustaining efficient T cell responses, the CD28 pathway is also involved in controlling immune tolerance. Co-stimulation of developing thymocytes by CD28 was shown to induce the expression of Foxp3 and promote the differentiation of regulatory T cells (Tregs; Tai et al., 2005). Furthermore, T cell activation in the absence of CD28 co-stimulation leads to a state of anergy characterized by dramatically reduced production of IL-2 and other effector cytokines upon subsequent TCR triggering (Schwartz, 1997). There is ample evidence that DCs utilize this mechanism to maintain tolerance to self. At steady state conditions, immature DCs present self-derived antigens accompanied by low levels of CD80/CD86 and therefore fail to supply specific T cells with adequate signal II, leading eventually to the deletion, anergy, or regulation of auto-reactive T cells that escaped thymic selection (Steinman and Nussenzweig, 2002). Thus, the CD80/CD86/CD28 pathway is as involved in promoting tolerance as in mediating immunity.

Since many immunogenic tumors lack expression of CD80 and CD86, it was postulated that tumor-infiltrating T cells would receive chronic TCR stimulation without co-stimulation leading to T cell anergy. This hypothesis was tested by inducing the expression of CD80/CD86 molecules on tumor cells prior to injection into mice. Forced expression of CD80/CD86 in tumor cells resulted into CD8^+^ T cell-dependent tumor rejection (Townsend and Allison, 1993). However, this method had barely any effect on pre-established tumors (Fallarino et al., 1997), implying that other pathways promoting immune tolerance toward established tumors are involved.

### CD40/CD40L PATHWAY

CD40 was the first co-stimulatory molecules to be identified from the tumor necrosis factor (TNF) receptor (TNFR) family. First discovered as B cell receptor, CD40 is also expressed by DCs, macrophages, epithelial cells, and even activated T cells. Its ligand (CD40L or CD154), a member of the TNF family, is expressed not only by activated T cells, but also by natural killer (NK) cells and plasmacytoid DCs (pDCs; Quezada et al., 2004). In addition to promoting humoral immunity by activating B cells, the CD40/CD40L pair is pivotal for cellular immunity as it mediates a dialog between T cells and DCs. Indeed, CD40 engagement on DCs was shown to activate NF-κB pathway (Quezada et al., 2004) and consequently inducing DC maturation (Caux et al., 1994) and enhancing DC longevity (Miga et al., 2001). Initially, CD40-induced maturation of DCs was suggested to be sufficient in licensing CD8^+^ cytotoxic responses (Schoenberger et al., 1998). However, further investigation in the CD40 pathway revealed that additional signals are necessary for optimal DC activation. CD40 cross-linking alone is not enough to induce IL-12 production, necessary for cytotoxic and Th1 responses, but DC pre-activation by microbial products followed by CD40 ligation dramatically increased IL-12 production (Schulz et al., 2000). This finding indicates that combined triggering of CD40 and PRRs, like Toll-like receptors (TLRs), is critical for DC licensing. The CD40-induced IL-12 also implies a central role for CD40/CD40L pathway in T cell differentiation, by favoring Th1 polarization. Blocking CD40/CD40L interactions lead to abrogated Th1 responses with reciprocal upregulation of Th2 cytokines (Hancock et al., 1998).

The adjuvant effect of CD40 ligation, reflected by DC activation, prompted the application of agonistic anti-CD40 antibodies for cancer therapy. Injecting agonistic anti-CD40 antibodies evoked cytotoxic T cell responses and eradicated the tumor in a mouse model of lymphoma (French et al., 1999). Furthermore, application of fully humanized anti-CD40 agonistic antibody resulted in objective partial responses in 14% of advanced solid tumor patients (Vonderheide et al., 2007). A similar approach was based on the administration of soluble CD40L, which was less efficient as it lead to partial responses in 6% of treated tumor patients (Vonderheide et al., 2001). More clinical trials applying CD40 ligation, singularly or in conjunction with other therapeutic modalities, were carried out and showed promising results (Khong et al., 2012).

Due to its activatory nature, the CD40/CD40L is decisive in regulating tolerance. It was shown that DCs derived from CD40-deficient mice conferred tolerance by priming IL-10 secreting Tregs (Martin et al., 2003). This effect on tolerance prompted investigating the possibility of exploiting CD40 blocking to enhance allograft survival. Although applying anti-CD40L antibodies as a monotherapy was able to block many effector mechanisms, it failed to induce sufficient allograft tolerance (Jones et al., 2000). However, combinations with other immunosuppressive therapies such as cytotoxic T lymphocyte (CTL)-associated antigen-4-immunoglobulin (CTLA-4-Ig; Larsen et al., 1996) and rapamycin (Li et al., 1998) were shown to result in long-term graft survival. Collectively, CD40/CD40L pathway, in conjunction with other pathways, is vital for initiating active immunity and regulating tolerance.

### ICOSL/ICOS PATHWAY

The inducible T cell co-stimulator (ICOS) was identified as the third member of the CD28/CTLA-4 family of co-stimulatory molecules (Hutloff et al., 1999). ICOS expression by T cells requires prior TCR activation and CD28 co-stimulation (McAdam et al., 2000). The ligand (ICOSL) is expressed by DCs (Wang et al., 2000), B cells, and a variety of non-hematopoietic tissues (Ling et al., 2000). ICOSL/ICOS pathway exerts its co-stimulatory effects on already activated T cells by supporting proliferation and cytokine production (Hutloff et al., 1999). Additionally, ICOS is proposed to play an important role in T cell polarization. Initially, ICOSL/ICOS was suggested to support Th2 responses. Blocking ICOSL/ICOS interactions was shown to block Th2-lead airway responses without influencing Th1-mediated inflammation (Coyle et al., 2000). Similarly, another study showed that the majority of T cells expressing ICOS *in vivo* co-produced Th2-type cytokines (Lohning et al., 2003). In contrast, disrupting ICOSL/ICOS pathway was found to inhibit Th1-mediated disorders like allograft rejection (Guo et al., 2002) and experimental allergic encephalomyelitis (Rottman et al., 2001). ICOS was shown to be involved driving Th17 responses (Park et al., 2005), further complicating the role of ICOSL/ICOS in T cell polarization. An attempt to resolve this controversy was by showing that engaging ICOS on activated T cells amplified the effector responses of these cells regardless of their polarized state (Wassink et al., 2004).

Benefiting of the activatory effect of ICOSL/ICOS pathway in the context of cancer therapy was evaluated. Induced ICOSL expression on tumor cells was demonstrated to promote tumor regression by inducing CD8 cytotoxicity (Liu et al., 2001). Nevertheless, this strategy was ineffective in case of weakly immunogenic tumors (Ara et al., 2003). Surprisingly, it was recently revealed that tumor cell-expressed ICOSL augments Treg activation and expansion within the tumor local environment (Martin-Orozco et al., 2010). This suggests that triggering ICOSL/ICOS pathway may not be the most optimal option for cancer treatment. On the contrary, blocking its ICOSL/ICOS-mediated suppression may be beneficial in cancer therapy.

The tolerogenic effect of ICOSL/ICOS pathway is not restricted to tumors, as there are indications of its involvement in maintaining immune tolerance. ICOS-deficient mice displayed reduced numbers of natural Tregs (nTregs), which may be owed to a decrease in survival and/or proliferation of these cells (Burmeister et al., 2008). Another indication of ICOS involvement in tolerance is the finding that ICOS triggering on T cells dramatically increased the production of the anti-inflammatory cytokine IL-10 (Hutloff et al., 1999). Consistently, high ICOS expression by T cells was selectively associated with the anti-inflammatory IL-10 (Lohning et al., 2003). These findings argue for targeting ICOSL/ICOS pathway to induce tolerance for therapeutic purposes. However, it is very important to clearly dissect the conditions under which this pathway induces activation or tolerance.

### CD70/CD27 PATHWAY

CD70 is another member of the TNF family of co-stimulatory molecules. Its ligand CD27 was identified first as a novel T cell differentiation antigen (van Lier et al., 1987). The contribution of CD27 to immunity was later recognized to be dependent on its binding partner CD70, which is expressed under the control of antigen receptors and TLRs in lymphocytes and DCs, respectively (Tesselaar et al., 2003). Similar to CD40, engaging CD27 induced the activation of NF-κB pathway (Akiba et al., 1998). The first indication of the co-stimulatory properties of the CD70/CD27 pathway was provided by triggering CD27, which augmented CD3-induced T cell proliferation (van Lier et al., 1987). This effect was later explained by promoting survival of newly stimulated T cells, in contrast to CD28 that prompts cell cycle entry and induces proliferation (Hendriks et al., 2003). This survival effect relies completely on IL-2 receptor signaling and the autocrine production of IL-2 (Peperzak et al., 2010).

The contribution of CD70/CD27 pathway to T cell polarization is debatable. CD8^+^ T cells from CD27 knockout mice maintained the capacity of differentiation into CTLs and interferon-gamma (IFN-γ) production, implying that CD27 is not involved in the development of cytotoxic CD8 responses (Hendriks et al., 2000). On the other hand, transgenic expression of CD70 on steady state immature DCs was found to break CD8^+^ tolerance and permit the differentiation of effector CD4^+^ and CD8^+^ cells from naïve precursors (Keller et al., 2008). Moreover, the murine CD8α^+^ DC subset was revealed to favor the differentiation of Th1 cells in a CD70-dependent and IL-12-independent mechanism (Soares et al., 2007). This is further supported by showing that human Langerhans cells (LCs), an epidermal subset of DCs, are capable of inducing CD8^+^ anti-viral responses in a CD70-dependent manner (van der Aar et al., 2011). A recent study also demonstrated that CD70/CD27 pathway impedes the differentiation of Th17 effector cells and attenuates accompanying autoimmunity in a mouse model of multiple sclerosis (Coquet et al., 2012). These findings imply that CD70 involvement in T cell polarization may depend on the type of DCs expressing CD70 and the type of stimuli to which these DCs are exposed.

The activatory effect of CD70/CD27 pathway can be exploited for anti-tumor therapy. Induced expression of both CD70 and CD40L by tumor cells was shown to impede tumor growth and initiate anti-tumor immunity (Couderc et al., 1998). Furthermore, the application of CD70, encoded in a vaccinia virus, was shown to confer protection against introduced tumors (Lorenz et al., 1999). Evidence for possible clinical benefit from mobilizing the CD70/CD27 pathway was provided by a recent clinical trial utilizing DCs expressing CD70, CD40L, and constitutively active TLR4 (TriMix-DC) in the treatment of metastatic melanoma patients. These TriMix-DCs were able to initiate a broad anti-tumor T cell response, resulting in prolonged progression-free survival (Van Nuffel et al., 2012). This paves the way for a novel strategy in cancer immunotherapy based on mobilizing the CD70/CD27 pathway.

Several reports have implicated CD70/CD27 pathway in autoimmunity. Elevated expression of CD70 by pathogenic T cells was observed in rheumatoid arthritis (Lee et al., 2007) and lupus erythematosus patients (Han et al., 2005). Moreover, blocking CD70/CD27 pathway seems to help ameliorating inflammation in mouse models of arthritis (Oflazoglu et al., 2009) and colitis (Manocha et al., 2009). However, the study reporting Th17 inhibiting effects of CD70 signaling (Coquet et al., 2012) may argue against the blockade of CD70/CD27 pathway, especially since Th17 effector cells are involved in various auto-inflammatory diseases.

### OX-40L/OX-40 PATHWAY

OX-40L and OX-40 belong to the TNF family and TNFR family, respectively. OX-40, also known as CD134, was first described on activated CD4^+^ T cells (Paterson et al., 1987). The expression of OX-40 is in fact restricted to recently antigen-activated T cells and not naïve or memory T cells, implying that it is specialized in delivering co-stimulation to activated T cells (Sugamura et al., 2004). The ligand, OX-40L (CD252), is expressed on DCs and macrophages, especially after TLR or CD40 ligation (Ohshima et al., 1997). Additionally, responding T cells express OX-40L themselves (Soroosh et al., 2006). Engagement of OX-40 on T cells promotes long-term survival by inducing the expression of the anti-apoptotic molecules Bcl-2 and Bcl-xL (Rogers et al., 2001). This study suggests that the differential expression kinetics of OX-40 and CD28, the latter being constitutively expressed by T cells, bares functional specialization. Whereas CD28 is essential for the initial priming of naïve T cells into effector T cells, OX-40 is crucial for the expansion (later proliferation) and survival of these effector cells.

Several studies have pointed out a central role for OX-40 in regulating the balance between Th1 and Th2 responses. Co-stimulating T cells through OX-40 was shown to induce IL-4 expression and inhibited IFN-γ production (Flynn et al., 1998). Furthermore, DC treatment with thymic stromal lymphopoietin (TSLP), known for its Th2 skewing properties, leads to the expression of OX-40L and the subsequent priming of Th2 cells (Ito et al., 2005). OX-40-favored Th2 response was proposed to be mediated by an initial induction of nuclear factor of activated T cells (NFAT) c1 in an IL-4 receptor-independent manner, followed by an IL-4 receptor-dependent effect on GATA-3 (So et al., 2006). However, it was shown later that DC-derived OX-40L maintained both Th2 and Th1 responses, owed to OX-40-enhanced survival of effector T cells regardless of their polarization (Jenkins et al., 2007). Thus, it seems that the role of OX-40/OX-40L in the differentiation of Th2 cells is restricted to promoting the survival of already established Th2 cells that differentiated under the effect of other DC-derived factors.

OX-40/OX-40L is also involved in controlling immune tolerance. The first evidence of this role is the expression of significant amounts of OX-40 on naturally occurring Foxp3^+^ Tregs. OX40 signaling appears to be dispensable for the development of nTregs, since this population exists in OX-40-deficient mice. However, OX-40 signaling is important for the survival of nTregs as OX-40-deficient mice displayed lower counts of this population of Tregs (Takeda et al., 2004). The effect of OX-40 triggering on the functions of nTregs remains controversial. Whereas one study showed that OX-40 signaling in CD4^+^ T cells render them resistant to suppression by nTregs (Takeda et al., 2004), another study reported abrogated suppression following OX-40 triggering on nTregs (Valzasina et al., 2005). Another mechanism by which OX-40L/OX-40 is assumed to contribute to tolerance regulation is by influencing the development of induced Tregs (iTregs). Under conditions promoting iTreg differentiation, OX-40 engagement on T cells was shown to inhibit Foxp3 expression by these T cells (So and Croft, 2007). Nevertheless, the surrounding environment during iTreg differentiation seems to determine the outcome of OX-40 signaling, which was reported to promote the expansion of iTregs if IL-4 and IFN-γ were absent from the milieu (Ruby et al., 2009). In conclusion, OX-40L/OX-40 appears to be central in maintaining the survival of T cells in general, but its influence on T cell functions requires further elucidation.

### 4-1BBL/4-1BB PATHWAY

4-1BB (CD137) is yet another member of the TNFR family. Its expression is induced on T cells following TCR activation (Pollok et al., 1993). The ligand, 4-1BBL of the TNF family, is expressed on activated APCs (Vinay and Kwon, 1998). Engagement of T cell 4-1BB was reported to induce IL-2 production independently of CD28, when accompanied by strong TCR signaling (Saoulli et al., 1998). Furthermore, 4-1BB interaction with its ligand was demonstrated to provide a co-stimulatory signal particularly to CD8^+^ T cells, enhancing proliferation, cytotoxicity (Shuford et al., 1997), and survival (Lee et al., 2002). Similar to other TNFR family members, 4-1BB enhanced survival is dependent on NF-κB activation, which in turn induces the two pro-survival molecules: Bcl-xL and Bfl-1 (Lee et al., 2002). When compared to co-stimulation with CD80/CD86, 4-1BBL appears to be more effective in driving CD8^+^ memory T cells into a fully differentiated effector state (Bukczynski et al., 2004). Furthermore, 4-1BB ligation was also shown to augment Th1 cytokines and suppress Th2 cytokines, implying a possible role for 4-1BB in T cell polarization (Kim et al., 1998). Collectively, these properties raised the interest in 4-1BBL/4-1BB pathway as potential therapeutic target especially in cancer therapy. Several studies demonstrated a beneficial effect of activating 4-1BB in inducing anti-tumor immunity and tumor regression thereafter (Driessens et al., 2009). Nevertheless, great caution should be taken before transferring these observations into clinical applications especially after reporting possible tolerogenic effects following 4-1BB triggering. Engaging 4-1BB by agonist antibodies was reported to ameliorate the severity of autoimmunity in murine models of experimental autoimmune encephalomyelitis (EAE) (Sun et al., 2002) and systemic lupus erythematosus (Foell et al., 2003), and to inhibit rejections of intestinal allografts in mice (Wang et al., 2003). These findings imply a link between 4-1BBL/4-1BB pathway and tolerance. Indeed, 4-1BB co-stimulation was shown to synergize with IL-2 in promoting nTreg expansion (Elpek et al., 2007). In an experimental model of rheumatoid arthritis, treatment with 4-1BB agonist antibodies inhibited disease progression, which was attributed to the induction of indoleamine 2,3-dioxygenase (IDO; Seo et al., 2004). Altogether, 4-1BBL/4-1BB pathway contributes to immunity and tolerance, allowing multiple therapeutic applications through this pathway.

### GITRL/GITR PATHWAY

Glucocorticoid-induced TNFR related gene (GITR) was first discovered as a dexamethasone-induced molecule in murine T cell hybridomas (Nocentini et al., 1997). The expression of the human ortholog was subsequently identified in human lymphocytes and shown to be independent of glucocorticoid treatment. Similar to the TNFR family members OX-40 and 4-1BB, GITR is only expressed on recently activated T cells, implying a role in promoting effector functions rather than involvement in initial priming of naïve T cells (Gurney et al., 1999). The GITR ligand (GITRL) is expressed by APCs and is upregulated upon activation (Tone et al., 2003). GITRL/GITR pathway provides co-stimulation to naïve T lymphocytes demonstrated by enhanced proliferation and effector functions in the setting of suboptimal TCR stimulation (Ronchetti et al., 2004). Additionally, GITR triggering promoted naïve T cell survival through the activation of NF-κB and mitogen-activated protein kinase (MAPK) pathways, though it was not sufficient to inhibit activation-induced cell death initiated by TCR signaling (Esparza and Arch, 2005). GITRL/GITR pathway does not seem to have an impact on T cell polarization. Although applying an agonist antibody against GITR initially enhanced Th2 responses in a mouse model of helminth infection, this effect was short lived and GITR-independent (van der Werf et al., 2011).

A role for GITRL/GITR pathway in immune tolerance was initially demonstrated by the constitutive expression of GITR on Tregs (Shimizu et al., 2002). Factually, Tregs isolated based on the expression of GITR could prevent the development of colitis induced in an adoptive transfer model (Uraushihara et al., 2003). However, engaging Treg-expressed GITR, by agonist antibodies, was shown to abrogate their suppressive capacity (Shimizu et al., 2002). In the beginning, this effect was interpreted by mere activation of Tregs upon GITR stimulation, but this explanation was underscored by the fact that Treg preincubation with anti-GITR did not cause the subsequent loss of suppression (Shimizu et al., 2002). Eventually, it was revealed that triggering GITR on effector T cells rendered them resistant to suppression by Treg (Stephens et al., 2004), providing a plausible explanation for the anti-tolerogenic effects of GITR stimulation. This postulates a model where APC-expressed GITRL would bind GITR on recently stimulated T cells allowing them to resist suppression. Simultaneously, GITR ligation on Tregs would allow their expansion and their subsequent domination at later stages of the immune response (Stephens et al., 2004).

Based on the activatory nature of GITRL/GITR pathway and its characteristic inhibition of tolerance, employing this pathway in cancer therapy was evaluated. The administration of an agonistic antibody against GITR has been shown to augment CD8 anti-tumor immunity (Cohen et al., 2006). In addition to mobilizing anti-tumor responses, triggering GITR was also shown to attenuate Treg-mediated suppression within the tumor (Ko et al., 2005), making GITRL/GITR a promising target for cancer therapy.

### LIGHT/HVEM PATHWAY

The TNFR family member herpes virus entry mediator (HVEM) was initially discovered as a receptor for herpes simplex virus (Montgomery et al., 1996). It is expressed on resting T cells, monocytes, and immature DCs. HVEM has multiple binding partners: LIGHT and lymphotoxin-α (LT-α) from the TNF superfamily; and CD160 and B and T lymphocyte attenuator (BTLA) from the Ig superfamily. HVEM interaction with these ligands creates a complex network of pathways, which collectively regulates adaptive immune responses (Ware and Sedy, 2011). In this section we will only focus on the co-stimulatory pathway resulting from LIGHT/HVEM interactions. LIGHT is expressed by immature DCs (Tamada et al., 2000a) and is induced upon activation on T cells, in contrast to HVEM (Morel et al., 2000). LIGHT/HVEM interaction was revealed to be required for DC-mediated allogenic T cell responses. Indeed, activating T cell HVEM enhanced T cell proliferation at suboptimal TCR stimulation conditions (Tamada et al., 2000a). Disrupted LIGHT/HVEM interaction was shown to result in inhibited T cell proliferation, further supporting the importance of this pathway in co-stimulation (La et al., 2002). Similar to other TNFR family members, HVEM mediates its effects by activating NF-κB pathway (Harrop et al., 1998). Interestingly, LIGHT/HVEM pathway can also contribute to T cell activation indirectly by inducing DC maturation, reminiscent of the role of CD40 in inducing DC maturation (Morel et al., 2001). LIGHT/HVEM pathway is also suggested to contribute to T cell polarization. T cells co-stimulated through HVEM displayed enhanced production of Th1 cytokines (Tamada et al., 2000b). Accordingly, LIGHT-deficient mice showed reduced IFN-γ levels, prolonging allograft survival in these mice (Ye et al., 2002). Due to the complexity of the signaling network of HVEM and LIGHT, reported findings should be interpreted as these observations may involve other pathways.

### TIM FAMILY

In addition to the CD28/B7 and TNFR/TNF co-stimulatory families, the recently identified TIM (T cell Ig domain and mucin domain) family is a new contributor to signal II. This family of genes was initially identified while searching for Th1-specific markers (Monney et al., 2002). In humans, three TIM family members: TIM1, TIM3, and TIM4 have been identified thus far. Mice posses an additional member: TIM2 (Kuchroo et al., 2008). In this section we will only focus on TIM3 and TIM4, which were reported to be expressed by DCs.

TIM3 was first discovered as a specific marker for Th1 cells (Monney et al., 2002), and was shown to induce the death of these cells by binding to its ligand galectin-9 (Zhu et al., 2005). TIM3 expression was also detected on DCs, and its ligation by galectin-9 induced the production of the inflammatory cytokine TNF-α. The absence of TIM3 signaling was shown to result in impaired TLR responsiveness, implying a synergistic relation between TIM3 and TLR signaling pathways (Anderson et al., 2007). Although TIM3 triggering on T cells and DCs leads to ERK (extracellular signal-regulated kinases) phosphorylation and IκBα degradation, different tyrosine phosphorylation patterns in T cells and DCs were detected, providing a plausible explanation for the differential effects of TIM3 between different cell types (Anderson et al., 2007). Thus far, interactions between DC-expressed TIM3 and T cell-expressed galectin-9 have not been investigated. However, previous findings prompt a model where DC-expressed TIM3 promotes inflammation and the differentiation of TIM3-expressing Th1 cells. IFN-γ-induced galectin-9 would interact with TIM3 from other T cells, inducing cell death and thereby self-limiting the immune response. Additionally, TIM3 is suggested to contribute to tolerance. A crucial role for TIM3 in clearing apoptotic cells by phagocytosis was recently revealed. Blocking this function resulted in inhibited cross-presentation of self-antigens and the development of auto-antibodies (Nakayama et al., 2009). In a completely different mechanism, TIM3 expressed by tumor-infiltrating DCs was shown to interact with the alarmin HMGB1, disturbing the recruitment of tumor cell-derived nucleic acids into DC endosomes, attenuating immune responses to these tumors (Chiba et al., 2012).

In contrast to the other members of the TIM family, TIM4 is exclusively expressed by APCs and not by T cells (Meyers et al., 2005). Through binding to TIM1 on T cells, TIM4 was shown to provide T cells with a co-stimulatory signal promoting T cell expansion, cytokine production, and survival. These effects were mediated by induced phosphorylation of the signaling molecules LAT (linker of activated T cells), Akt, and ERK1/2 in stimulated T cells (Rodriguez-Manzanet et al., 2008). Notably, the strength of TIM4 signal is decisive in determining the stimulatory effect, as weak TIM4 signaling inhibits T cell proliferation instead of potentiating it (Meyers et al., 2005). Similarly, TIM4 was shown to inhibit the proliferation of naïve T cells, which lack the expression of TIM1 (Mizui et al., 2008). These data imply that TIM4 has at least two binding partners: an activating ligand (TIM1) and an inhibitory one to be identified. Through these ligands, TIM4 exerts bimodal regulation of immune responses. Analogous to TIM3, the role of TIM4 in regulating immunity is also evident through mediating the engulfment of apoptotic cells. *In vivo* blocking of TIM4 resulted in the development of auto-antibodies (Miyanishi et al., 2007).

### ADHESION MOLECULES PROVIDING CO-STIMULATORY SIGNALS

Leukocyte adhesion and detachment from other cells is tightly regulated by adhesion molecules. A specific set of these molecules is involved in regulating DC/T cell interactions. This set includes the following molecules: intercellular adhesion molecule 1 (ICAM-1) and lymphocyte function-associated antigen-3 (LFA-3), expressed by DCs, and their respective ligands LFA-1 and CD2, expressed by T cells. The seminal discovery of the involvement of LFA-1 in mediating T cell functions prompted a hypothesis that LFA-1 would act by enhancing adhesion and thereby increasing the range of avidities that can promote antigen recognition (Springer et al., 1982). Subsequently, ICAM-1 was identified as the ligand of LFA-1 (Rothlein et al., 1986). LFA-1 ligation by ICAM-1 was shown to induce proliferation of TCR-stimulated T cells in an IL-2-dependent mechanism, proposing that ICAM-1/LFA-1 interaction as a co-stimulatory pathway (Van Seventer et al., 1990). In addition to co-stimulation, ICAM-1/LFA-1 interaction stabilizes the immunological junction (Bleijs et al., 2001) and the ICAM-1/LFA-1 pathway appears to contribute to T cell differentiation as repeated T cell stimulation with ICAM-1 promoted IFN-γ production by these cells (Semnani et al., 1994).

Moreover, blocking ICAM-1/LFA-1 interactions during T cell stimulation drastically increased Th2 cytokines (Salomon and Bluestone, 1998). More recently, ICAM-1/LFA-1 interaction during CD8^+^ T cell priming was demonstrated to be essential for the establishment of effective T cell memory (Scholer et al., 2008). The effects of the ICAM-1/LFA-1 pathway are believed to result from influencing multiple cellular signaling cascades. LFA-1 was found to interact with the transcriptional co-activator JAB1, implying an influence on c-Jun-driven transcription (Bianchi et al., 2000).

In parallel, T cell CD2 interaction with its ligand LFA-3 was recognized for contributing to T cell activation by strengthening the adhesion between T cells and APCs and thereby enforcing TCR contact with its ligands (Davis and van der Merwe, 1996). Moreover, CD2 signaling was also shown to restore responsiveness in anergized human T cells (Boussiotis et al., 1994). CD2 blocking *in vivo* was revealed to induce T cell unresponsiveness, further supporting the notion that LFA-3/CD2 pathway contributes to immune activation (Xu et al., 2004). Conversely, specific mobilization of LFA-3/CD2 interactions was demonstrated to induce, single handedly, non-proliferating Tregs secreting high amounts of IL-10 (Wakkach et al., 2001). In light of these contradictions, further characterization of the role of LFA-3/CD2 co-stimulatory pathway is required.

## CO-INHIBITORY MOLECULES

### CD80/CD86/CTLA-4 PATHWAY

Cytotoxic T lymphocyte-associated antigen-4 (CD152) is a CD28 homolog that was discovered in 1987 (Brunet et al., 1987). The closely related structures of these two molecules suggest overlapping functional qualities. Indeed, CTLA-4 binds to CD80 and CD86, though at greater affinities. However, CTLA-4 was the first described co-stimulatory molecule with inhibitory effects in a stark contrast to the activatory properties of CD28 (Linsley et al., 1991b). The effects of CTLA-4 include inhibition of proliferation, cell cycle progression, and IL-2 synthesis (Walunas et al., 1996). Additionally, CTLA-4 seems to have an influence on T cell polarization. T cells lacking CTLA-4 expression were shown to adopt a Th2 phenotype (Bour-Jordan et al., 2003). Furthermore, neutralizing CTLA-4 signaling in T cells was recently shown to enhance IL-17 production and promote the differentiation of Th17 cells (Ying et al., 2010).

The prominent role of CTLA-4 in tolerance is clearly demonstrated by CTLA-4-deficient mice, which succumb at 3–4 weeks of age to massive lymphoproliferative disease (Tivol et al., 1995). Furthermore, the suppressive functions of naturally occurring Tregs, which constitutively express CTLA-4, were dependent on CTLA-4 signaling (Read et al., 2000), corroborating its role in tolerance. CTLA-4 contribution to tolerance is postulated to arise from controlling T cell responses in an intrinsic or extrinsic manner (Rudd et al., 2009). First, CTLA-4 antagonizes the CD28 stimulatory signaling by competing with CD28 on binding to CD80/CD86. Interestingly, CTLA-4 expression on cells is induced in a CD28-dependent mechanism (Alegre et al., 1996), implying that CTLA-4 serves as an internal checkpoint that prohibits excessive stimulation by CD28. Extrinsic inhibitory effects of CTLA-4 are suggested to be exerted through different mechanisms. CTLA-4 molecules expressed by Tregs were shown to engage CD80/CD86, expressed by DCs, promoting the activity of IDO. The modified catabolic properties of DCs lead to localized deprivation of tryptophan and thereby reduced T stimulatory capacity of these DCs (Fallarino et al., 2003). Another suggested mechanism for the extrinsic effects of CTLA-4 was demonstrated by the capacity of CTLA-4 to capture CD86, expressed by APCs, internalize it for ensuing degradation in a process called *trans*-endocytosis (Qureshi et al., 2011). Tregs were also observed to suppress T cells by establishing a direct interaction through CTLA-4, which binds to CD80 and CD86 expressed by those T cells (Taylor et al., 2004). Finally, unstimulated T cells were revealed to produce a soluble form of CTLA-4, which may possibly convey the inhibitory effects to other cells (Magistrelli et al., 1999). Collectively, CTLA-4 is unequivocally vital for tolerance.

Due to its role in maintaining tolerance, blocking CTLA-4 interaction with CD80 and CD86 was postulated to promote anti-tumor immunity. Indeed, *in vivo* administration of blocking antibodies against CTLA-4 resulted into effective anti-tumor immunity and tumor rejection (Leach et al., 1996). Nevertheless, CTLA-4 blockade efficacy in tumor therapy was correlated with the stage and immunogenicity of the tumor. At early stages small tumors were sensitive to the effects of CTLA-4 blockade (Shrikant et al., 1999), whereas advanced tumors were resistant due to the strongly tumor-induced T cell tolerance (Sotomayor et al., 1999). In an attempt to circumvent this hurdle, anti-CTLA-4 blocking antibodies were tested in combination with other therapeutic modalities. Combined anti-CTLA-4 application and Treg depletion resulted in maximal tumor rejection, which was dependent on the expansion of tumor-specific CD8^+^ T cells (Sutmuller et al., 2001). Those promising experimental observations lead to the development of two fully human anti-CTLA-4 antibodies: ipilimumab (Bristol-Myers Squibb, New York, NY, USA) and tremelimumab (Pfizer, New York, NY, USA). Early clinical trials in metastatic melanoma and ovarian carcinoma patients demonstrated that blocking CTLA-4 resulted in extensive tumor necrosis with lymphocyte and granulocyte infiltrates in a large number of patients (Hodi et al., 2003). Further large scale clinical trials have shown irrefutable evidence of the efficacy of anti-CTLA-4 antibodies, leading eventually to FDA approval of these antibodies (Kirkwood et al., 2012). Despite its novelty, this therapeutic strategy is challenged by autoimmune complications resulting from the administration of anti-CTLA-4 antibodies (Sanderson et al., 2005).

The tolerogenic effects arising from CTLA-4 engagement with CD80/CD86 can also be utilized for inducing tolerance toward transplanted tissues. This notion has been supported by observations in animal experimental models. Administration of recombinant CTLA-4-Ig fusion protein after renal or cardiac transplantation enhanced allograft acceptance and reduced inflammatory responses (Azuma et al., 1996). This led to the development of humanized CTLA-4-Ig (Belatacept). Kidney transplantation patients receiving Belatacept showed reduced allograft rejection and maintained better renal functions, compared to patients receiving cyclosporine. These findings resulted in gaining FDA approval for using Belatacept for the prevention of acute rejection post-renal transplant (Vincenti et al., 2011).

### PD-L1/PD-L2/PD-1 PATHWAY

Programed cell death-1 (PD-1) is another member of the CD28 family that is expressed by activated T and B cells (Agata et al., 1996). Two ligands were identified to interact with PD-1: PD-L1 (Dong et al., 1999) and PD-L2 (Latchman et al., 2001). Those ligands are characterized by differential expression patterns. PD-L1 is constitutively expressed and further enhanced on activated lymphocytes, including Tregs and DCs. It is also expressed by a wide variety of non-hematopoietic cell types including the vascular endothelial cells, neurons and pancreatic islet cells. In contrast, PD-L2 expression is restricted to DCs and macrophages under certain conditions (Greenwald et al., 2005). Interestingly, PD-L2 displays three times higher binding affinity to PD-1 in comparison to PD-L1, which on the other hand was also identified to bind to CD80 (Butte et al., 2007). The varying binding and expression properties of PD-L1 and PD-L2 suggest distinct functions in regulating T cell responses. Along with its ligands PD-1, is recognized for its vital role in regulating adaptive immune responses (Sharpe et al., 2007). Indeed, triggering of PD-1 by one of its ligands during TCR signaling can block T cell proliferation, cytokine production and cytolytic activity, and impair T cell survival (Riley, 2009). The intracellular domain of PD-1 contains an immunoreceptor tyrosine-based inhibitory motif (ITIM) as well as an immunoreceptor tyrosine-based switch motif (ITSM), which are phosphorylated upon ligand engagement. Subsequently protein phosphatases, such as Src homology phosphatase-1 (SHP-1) and SHP-2, are recruited to TSM where they are activated and inhibit proximal TCR signaling events by dephosphorylating key intermediates in the TCR signaling cascade (Chemnitz et al., 2004). Similar to CTLA-4, triggering PD-1 limits glucose metabolism and Akt activation, albeit through different mechanisms (Chemnitz et al., 2004). Consistently, a recent study also demonstrated that PD-1 exerted its inhibitory effects by affecting Akt and Ras pathways and thereby inhibiting cell cycle progression and T cell proliferation (Patsoukis et al., 2012).

The first indication of the importance of PD-1 in immune tolerance came from PD-1-deficient mice, which developed strain-specific autoimmunity. The absence of PD-1 caused the development of cardiomyopathy secondary to the production of auto-antibodies against cardiac troponin in BALB/c mice (Nishimura et al., 1999), while C57BL/6 developed a lupus-like autoimmune disease (Nishimura et al., 2001). In humans, polymorphisms in the PD-1 gene were also associated with susceptibility to several autoimmune diseases including systemic lupus erythematosus (Prokunina et al., 2002), type I diabetes (Nielsen et al., 2003), and multiple sclerosis (Kroner et al., 2005). These observations were supported by functional studies demonstrating the contribution of the PD-L1/PD-L2/PD-1 pathway to central tolerance. In the thymus, interactions between PD-1, expressed by CD4^-^CD8^-^ thymocytes, and PD-L1 broadly expressed in the thymic cortex, were deemed crucial in regulating positive selection (Nishimura et al., 2000). PD-1 was also shown to participate in thymic negative selection (Blank et al., 2003). Gene expression profiling studies of central tolerance in non-obese diabetic (NOD) mice also implicated PD-1 and PD-L1 in central tolerance (Zucchelli et al., 2005). PD-L1/PD-L2/ PD-1 pathway also contributes to peripheral tolerance through multiple mechanisms. Self-reactive CD8^+^ T cells lacking PD-1 display increased responsiveness to self-antigens presented by resting DCs, suggesting that DC-expressed PD-L1 and PD-L2 may control T cell activation (Probst et al., 2005). PD-L1/PD-L2/PD-1 pathway can also regulate reactivation, expansion, and functions of effector T cells (Keir et al., 2006). Additionally, PD-1 triggering of TCR-stimulated, transforming growth factor-beta (TGF-β)-treated T cells profoundly enhanced the *de novo* generation of Foxp3^+^ Tregs from CD4^+^ naïve precursors. Further engagement of PD-L1 on the iTregs sustained Foxp3 expression and enhanced the suppressive capacity of these cells (Francisco et al., 2009). Consistently, PD-L1 was shown to mediate the effects of the immune suppressant vitamin D (VitD). DCs treated with VitD were shown to induce IL-10 producing Tregs in a PD-L1-dependent mechanism (Unger et al., 2009). Interactions between PD-1 and PD-L1 are also proposed to maintain tolerance by modifying DC–T cell contact. PD-1 ligation was shown to inhibit the TCR-induced stop signals, disrupting the stable DC–T cell contact and subsequently allowing tolerized T cells to move freely and prohibiting clustering around antigen-bearing DCs (Fife et al., 2009). Another plausible mechanism for PD-L1/PD-L2/PD-1 pathway-induced tolerance is that PD-L1 expressed by Tregs would engage PD-1 expressed by DCs and modulate DC function and thereby impeding immune responses (Francisco et al., 2010).

The inhibitory effects of PD-L1/PD-L2/PD-1 pathway can be hijacked by tumors to evade anti-tumor immune responses. PD-L1 expression has been confirmed on many tumors including glioblastoma and melanoma as well as cancers of the head and neck, lung, ovary, colon, stomach, kidney, and breast. High expression PD-L1 levels by tumor cells, tumor-infiltrating lymphocytes, or both associated with aggressive tumor behavior, poor prognosis, and elevated risk of mortality (Zang and Allison, 2007). Moreover, DCs generated from peripheral blood of ovarian cancer patients displayed high levels of PD-L1, prompting impaired T cells responses, which were restored by blocking PD-L1/PD-1 interactions (Curiel et al., 2003). *In vivo*, forced PD-L1 expression by squamous cancer cells rendered them resistant to T cell-mediated immunity. This resistance, however, was broken upon treatment with anti-PD-L1 blocking antibodies (Strome et al., 2003). A recent study also revealed that platinum based chemotherapeutics enhanced anti-tumor T cell responses by disrupting PD-L2/PD-1 interactions through reducing PD-L2 levels on both DCs and tumor cells (Lesterhuis et al., 2011). These experimental observations prompted the development of humanized anti-PD-1 and anti-PD-L1 antibodies for clinical application. Early stage clinical trials with these antibodies demonstrated clinical activity, which was characterized by durability accompanied with minimal side effects (Zitvogel and Kroemer, 2012).

There is also evidence that viral infections can make use of PD-L1/PD-L2/PD-1 pathway. Animal models of chronic viral infections had elevated PD-1 expression on exhausted viral antigen-specific T cells. The activity of these T cells was restored following PD-L1 blocking, suggesting a novel strategy for combating chronic viral infections (Barber et al., 2006).

In line with its inhibitory role, PD-L1/PD-L2/PD-1 pathway can be harnessed for the induction of tolerance when needed. Administration of recombinant PD-L1-Ig, with agonistic effect for PD-1, prolonged the survival of cardiac allografts in mice (Ozkaynak et al., 2002). Furthermore, PD-L1 expression on murine liver allografts is central for spontaneous tolerance (Morita et al., 2010).

### B7-H3 PATHWAY

B7-H3 belongs to the B7 family of co-stimulatory molecules. Similar to other Ig superfamily members, B7-H3 is a transmembrane molecule. It possesses a short cytoplasmic tail with no known signaling domain. B7-H3 is expressed on a wide a variety of tissues and tumor cell lines. However, its expression on leukocytes is only detectable following stimulation. B7-H3 expression can be induced on DCs and monocytes by inflammatory cytokines, whereas a combination of phorbol myristate acetate and ionomycin can induce it on T cells. B7-H3 was shown to bind a receptor expressed by activated T cells. This receptor is distinct from CD28, CTLA-4, ICOS, and PD-1 and yet to be identified (Chapoval et al., 2001). Triggering receptor expressed on myeloid cells (TREM)-like transcript 2 (TLT-2), constitutively expressed by CD8^+^ T cells and activation-induced on CD4^+^ T cells, was proposed to be the binding partner of B7-H3 (Hashiguchi et al., 2008). However, this was strongly refuted by another study providing evidence of non-existing interaction between B7-H3 and TLT-2 (Leitner et al., 2009). Initially, B7-H3 was suggested to be a positive co-stimulatory molecule that induces T cell proliferation, IFN-γ production and CTL generation in humans (Chapoval et al., 2001). Nevertheless, this was contradicted by another study demonstrating that B7-H3 is a potently inhibited T cell stimulation under different conditions and regardless of the stimulation status of the T cells in question (Leitner et al., 2009). This is corroborated by data from murine studies where applying an agonistic fusion protein, B7-H3-Ig, was shown to inhibit proliferation, IL-2 and IFN-γ production of TCR-stimulated T cells. This inhibitory effect was demonstrated by exacerbated airway inflammation in B7-H3-deficient mice compared to wild type counterparts (Suh et al., 2003). Moreover, blocking B7-H3 caused enhanced T cell proliferation *in vitro* and worsened EAE *in vivo*. This effect may be explained by the inhibitory influence of B7-H3 signaling over NF-κB, NFAT, and AP-1 that are involved in regulating T cell activation (Prasad et al., 2004). Notably, the effects of B7-H3 were overridden by CD28 co-stimulation, implying that B7-H3 functions optimally in the absence of co-stimulation (Suh et al., 2003). Of interest, tumors are suggested to hijack the B7-H3 to evade anti-tumor immune responses. This is demonstrated by increased disease severity when cancer cells upregulated B7-H3 expression (Hofmeyer et al., 2008). Collectively, further characterization of the B7-H3 pathway is required to resolve functional discrepancies, which may be explained by the existence of two receptors for B7-H3 with opposite functions, yet to be identified.

### B7-H4 PATHWAY

B7-H4 is the last among the B7 family members that was identified. Unlike other B7 family members, which are type I membrane molecules, B7-H4 is characterized by a glycosylphosphatidylinositol (GPI) domain that links to the cell membrane (Prasad et al., 2003). In humans, B7-H4 mRNA was detected in a variety of tissues. However, immunohistochemical analysis did not reveal any B7-H4 protein expression by these tissues. Likewise, no B7-H4 expression could be detected on freshly isolated T cells, B cells, monocytes, and DCs, but it was induced after activating these cells *in vitro*. The ligand of B7-H4 has not been identified yet, but it is suggested to be expressed by stimulated T cells and to be distinct from other CD28 family members (Sica et al., 2003). B7-H4 is widely regarded as a co-inhibitory molecule. Indeed, treatment of TCR-stimulated T cells by a fusion B7-H4-Ig protein resulted in inhibited T cell proliferation and cytokine production, an effect that required B7-H4 cross-linking (Sica et al., 2003). The inhibitory effects of B7-H4 are proposed to arise from arrested cell cycle progression in T cells (Sica et al., 2003), and impaired induction of JunB, known for its role in inducing IL-2 production in activated T cells (Prasad et al., 2003). A recent study also showed that B7-H4 signaling inhibits phosphorylation of MAP kinases, ERK, p38, Jun N-terminal kinase (JNK), and Akt, usually elicited upon TCR triggering of T cells (Wang et al., 2012a).

In line with *in vitro* findings, mice suffering from graft versus host disease demonstrated prolonged survival upon the *in vivo* application of B7-H4-Ig (Sica et al., 2003). Expectedly, *in vivo* administration of an antagonizing antibody against B7-H4 blocked the inhibitory effect of B7-H4 pathway and led to accelerated disease development in a mouse model of EAE (Prasad et al., 2003). Furthermore, B7-H4-deficient mice showed better control of *Leishmania major* infection as Th1 responses were augmented in these mice (Suh et al., 2006). B7-H4 deficiency also enhanced neutrophils-mediated immunity, implying that B7-H4 may have a role in regulating innate immunity too (Zhu et al., 2009). In addition to its role as a co-inhibitory molecule, B7-H4 seems to mediate the effect of Tregs. It was shown that Tregs, but not conventional T cells, induce high levels of IL-10 production by APCs and consequently trigger B7-H4 expression that renders these APCs immunosuppressive (Kryczek et al., 2006a). The overall tolerogenic effect of B7-H4 can be exploited by tumors to evade immune responses. B7-H4 expression was reported for several tumors including lung cancer, ovarian cancer (Choi et al., 2003), gastric cancer (Jiang et al., 2010), and tumor-associated macrophages (Kryczek et al., 2006b). Blockade of B7-H4 on these macrophages was actually effective in reversing their suppressive effect and restored anti-tumor T cell immunity (Kryczek et al., 2006b). Additionally, manipulating B7-H4 pathway has potential in the field of transplantation. A recent study showed that B7-H4 expression was shown to prolong islet allograft survival in mice (Wang et al., 2012b). Thus, the B7-H4 pathway serves as an interesting therapeutic target in different diseases, though several aspects of this pathway remain elusive.

### HVEM/BTLA/CD160 PATHWAY

As mentioned earlier, the molecules HVEM, BTLA, CD160, and LIGHT interact directly with each other forming a complex pathway network regulating adaptive immune responses. HVEM, expressed by immature DCs, can provide negative co-stimulatory signals through binding to its ligands BTLA and CD160 on T cells (Ware and Sedy, 2011). BTLA belongs to the Ig superfamily and is a structural homolog of CTLA-4 and PD-1. It is also a transmembrane glycoprotein that can be phosphorylated on tyrosines located in conserved cytoplasmic ITIM motif (Watanabe et al., 2003). T cell expression of BTLA was shown to be very low on naïve cells. However, it is upregulated upon antigen-stimulation peaking at day 2 and declining around day 7 post-stimulation. This expression can be retrieved upon secondary stimulation of activated T cells. Interestingly, anergic T cells and Th1 cells demonstrated high BTLA expression unlike Th2 cells and Tregs that have low BTLA expression (Hurchla et al., 2005). The unique BTLA expression pattern and expression kinetics indicate that BTLA may interfere at certain stages of T cell activation with specificity to certain types of effector T cells.

Herpes virus entry mediator delivers its inhibitory signal to T cells by binding to BTLA, which induces the phosphorylation of its ITIM domain and the recruitment of SHP-2, leading to attenuated antigen-driven T cell activation (Sedy et al., 2005). In addition to inhibiting T cell responses, there is evidence that HVEM/BTLA pathway promotes T cell survival in a mechanism dependent on NF-κB activation (Cheung et al., 2009). Interestingly, BTLA was also shown to mediate Treg suppression by interacting with HVEM expressed by Tregs. This was supported by showing that Tregs from HVEM-deficient mice had lower suppressor activity and that wild type Tregs failed to suppress effector T cells from BTLA-deficient mice (Tao et al., 2008). The inhibitory effects of BTLA are also observed *in vivo*. In an EAE model, BTLA-deficient mice displayed increased severity and persistence of disease when compared with wild type controls (Watanabe et al., 2003). BTLA deficiency was also reported to exacerbate allergic airway inflammation (Deppong et al., 2006) and to cause the development of auto-antibodies leading to a hepatitis-like syndrome with advancing age (Oya et al., 2008). Moreover, a single-nucleotide polymorphism (SNP) in the ITIM region of BTLA was reported to associate with increased susceptibility to rheumatoid arthritis (Lin et al., 2006). Another study also revealed an association between another BTLA SNP and rheumatoid arthritis, but not with systemic lupus erythematosus or Sjogren’s syndrome (Oki et al., 2011). Similar to B7-H3 and B7-H4, the inhibitory effects of BTLA can be exploited by tumors to evade immunity. Melanoma-specific CD8^+^ T cells were shown to persistently express BTLA. Interrupted BTLA signaling, achieved by applying CpG oligonucleotide vaccine formulations, lead to functional recovery of melanoma-specific CD8^+^ T cells (Derre et al., 2010).

Herpes virus entry mediator can also interact with CD160, a GPI anchored membrane molecule that is mainly expressed by CD8^+^ T cells and activated CD4^+^ T cells. Cross-linking CD160 with a specific antibody on stimulated T cells was shown to strongly inhibit T cell proliferation and cytokine production. Similarly, the inhibitory effect of CD160 was also elicited by binding to its ligand HVEM (Cai et al., 2008). Although both BTLA and CD160 bind to the cysteine-rich domain-1 (CRD-1) of HVEM with comparable affinity, CD160 dissociates from HVEM at a slower rate compared to BTLA. Moreover, mutagenesis study of HVEM revealed that CD160 has a distinct binding site on HVEM, albeit overlapping with BTLA (Kojima et al., 2011). Those differences between CD160 and BTLA, though subtle, suggest that these molecules do not have redundant functions. Further delineation of the elusive HVEM/CD160 pathway and its functional implications are required to unravel its specific role in regulating immune responses.

### ILT3 AND ILT4/HLA-G PATHWAYS

The inhibitory receptor Ig-like transcript-3 (ILT3; Cella et al., 1997) and ILT4 (Colonna et al., 1998), both expressed by monocytes, macrophages, and DCs, belong to a family of Ig-like inhibitory receptors that are closely related to the killer cell inhibitory receptors. Both ILT3 and ILT4 were shown to transmit signal through a long cytoplasmic tail containing ITIM motifs, which inhibit cell activation by recruiting the protein phosphatase SHP-1 (Cella et al., 1997; Colonna et al., 1998). In the case of ILT3, the extracellular region consists of two Ig-like domains, which are speculated to contain the putative binding site of the yet to be identified ILT3 ligand (Cella et al., 1997). On the other hand, the binding partner of ILT4 was shown to be the MHC class I molecule human leukocyte antigen G (HLA-G; Colonna et al., 1998). In addition to triggering an inhibitory signal, ILT3 cross-linking was shown to lead to its internalization and delivery into an antigen presenting compartment, suggesting a role in antigen processing (Cella et al., 1997). DC expression of ILT3 and ILT4 was shown to be induced under the effect of CD8^+^CD28^-^ alloantigen-specific T suppressor cells (Chang et al., 2002). Immature monocyte-derived DCs (MoDCs) also upregulated ILT3 and ILT4 expression upon treatment with either IL-10 or/and IFN-α (Manavalan et al., 2003). VitD treatment only induced ILT3 expression in MoDCs (Manavalan et al., 2003) and primary human blood BDCA1^+^ DCs (Chu et al., 2012). Expectedly, ILT3 expression, by both MoDCs and pDCs, was downregulated following activation (Ju et al., 2004).

Tolerogenic DCs over-expressing ILT3 or ILT4 demonstrated impaired NF-κB activation and consequently reduced transcription capacity of NF-κB-dependent co-stimulatory molecules (Chang et al., 2002). Those DCs were shown to be capable of transforming alloreactive effector T cells into antigen-specific Tregs (Manavalan et al., 2003). Similarly, triggering ILT4 by HLA-G tetramers was shown to impair maturation and T cell stimulatory capacity of human DCs (Liang and Horuzsko, 2003). Interestingly, ILT3 was shown to maintain its T cell inhibitory effect when it was expressed as soluble ILT3-Fc that lacks ILT3’s cytoplasmic tail, indicating that ILT3 delivers its inhibitory signal by binding to its partner on activated T cells (Kim-Schulze et al., 2006). Recently it was shown that ILT3 capacity to convert T cells into suppressive cells is dependent on BCL6 signaling in these T cells (Chang et al., 2010). ILT3 is also proposed to be important for controlling inflammation, as silencing ILT3 expression in DCs enhances TLR responsiveness, which is reflected by enhanced secretion of inflammatory cytokines such as IL-1α, IL-1β, IL-6, and IFN-α. ILT3-silenced DCs could also attract more lymphocytes by secreting high levels of the chemokines CXCL10 and CXCL11 in response to TLR ligation. Eventually, impaired ILT3 expression in DCs rendered them more stimulatory for T cells, which also secreted higher levels of cytokines like IFN-γ and IL-17 (Chang et al., 2009). Another suggested mechanism by which both ILT3 and ILT4 contribute to tolerance is by possibly mediating the effects of IDO. DCs cultured in tryptophan-deprived local environment upregulated the expression of ILT3 and ILT4, favoring the development of Foxp3^+^ Tregs (Brenk et al., 2009). Finally, ILT4 was shown to be central for the development of type I Tregs, induced by IL-10-treated DCs (Gregori et al., 2010).

The effects of ILT3 and ILT4/HLA-G pathways are also evidenced *in vivo*. Immune modulation exerted by ILT4/HLA-G interactions is believed to mediate maternal tolerance toward the semi allogenic fetus (Hunt et al., 2005). Moreover, *in vivo* treatment with VitD was shown to upregulate the expression of ILT3 on DCs in healing psoriatic lesions. Nevertheless, ILT3 was revealed to be dispensable for the induction of Tregs and completely overridden by the inhibitory effects of VitD (Penna et al., 2005). Consistently, maternal VitD intake during pregnancy was found to enhance ILT3 and ILT4 gene expression levels in cord blood, pointing out a plausible mechanism for early induction of immune tolerance (Rochat et al., 2010). Enhanced ILT3 and ILT4 levels were also observed at an early stage of venom-specific immunotherapy, implying a possible role in inducing tolerance toward allergic reactions (Bussmann et al., 2010). Owed to its inhibitory effects, ILT3 is suggested to be employed by tumors as a mean of evading anti-tumor immunity. Indeed, soluble ILT3 protein was found at high levels in the serum of patients with melanoma, and carcinomas of the colon, rectum, and pancreas produce. This soluble ILT3 was active in inducing suppressor CD8^+^ T cells that block anti-tumor immunity, which was restored upon blocking or depleting ILT3 (Suciu-Foca et al., 2007). A similar mechanism is also utilized by viruses, as demonstrated by a point mutation in one of HIV Gag epitopes that increased binding to ILT4 and consequently programed myelomonocytic cells to become tolerogenic (Lichterfeld et al., 2007). The inhibitory effects of ILT3 can also be harnessed for allograft acceptance. Indeed, soluble recombinant ILT3-Fc was shown to suppress T cell-mediated rejection of allogenic islet transplants in mice (Vlad et al., 2008). In correlation to its inhibitory effect, blood monocytes during multiple sclerosis relapses demonstrated lower ILT3 expression, which was restored upon treatment with IFN-β, unraveling a plausible therapeutic target in the treatment of multiple sclerosis (Jensen et al., 2010). Similarly, a SNP in the ILT3 extracellular region was correlated with low surface expression and increased serum cytokine levels in lupus patients (Jensen et al., 2012).

## CONCLUDING REMARKS AND FUTURE PROSPECTS

Since the identification of the CD80/CD86/CD28 classical co-stimulatory pathway, the concept of DC-derived signal II was dramatically expanded to accommodate the ever increasing number of newly discovered co-stimulatory and co-inhibitory pathways. An increasing body of reports reflects the complexity of these pathways and implies possible interactions to form a sophisticated network controlling adaptive immune responses. The existence of multiple co-stimulatory and co-inhibitory pathways postulates for overlapping functions. Nevertheless, this notion of redundancy should be considered carefully. The components of these pathways have distinct expression patterns and kinetics, which means that these pathways are not simultaneously operative. In addition, mobilizing these pathways can trigger distinct signaling cascades and thereby leading to variable outcomes.

Dendritic cell expression of co-stimulatory and co-inhibitory molecules is dictated by several factors. The specific type of DC is a major determinant of this expression. In humans, DCs are classified into groups based on origin, specific expression of certain surface markers, and functional properties. For example, human blood DCs are divided into two major subsets: pDCs and myeloid DCs (myDCs). The latter can be further divided into three subsets: BDCA1^+^ DCs, BDCA3^+^ DCs, and CD16^+^ DCs. In parallel, skin DCs are also classified into epidermal LCs, dermal CD1a^+^ DCs, and dermal CD14^+^ DCs. Similar classification can be expected in other tissue-resident DCs. Most of the findings concerning co-stimulatory and co-inhibitory molecules in humans were based on experiments performed on the *in vitro* generated MoDCs, which serve as a great tool for delineating immunological functions and mechanisms. However, there are strong indications of differential expression of co-stimulatory and co-inhibitory molecules among different DC subsets. These variations can be partially related to the intrinsic qualities of every DC subset. For instance, pDCs and LCs lack the expression of TLR4, and consequently they are not able to upregulate CD80 and CD86, observed in other subsets in response to lipopolysaccharide (LPS).

Another central determinant of co-stimulatory and co-inhibitory molecules expression by DCs is the type of stimulus, to which DCs are exposed. As mentioned earlier, DCs respond to pathogen stimulation by upregulating CD80 and CD86. However, there are indications that certain co-stimulatory molecules are strictly expressed upon activation with a specific class of pathogens. A clear example is CD70 expression by LCs upon TLR3 triggering by double-stranded RNA derived from viruses, granting LCs advantage in eliciting strong anti-viral CD8^+^ T cell responses. Although dermal DCs and MoDCs express TLR3, they do not upregulate CD70 in response to double-stranded RNA, implying a combined effect of the type of stimulus and the type of DC in inducing CD70 expression. Similarly, pDC stimulation with CpG B, a TLR9 ligand, induced the expression of CD70, which was not observed using another type of stimulation or in other DC subsets (Shaw et al., 2010). Another example demonstrating the effect of pathogenic stimulation is the upregulation of OX40L only upon exposure to the soluble egg antigen from the parasite *Schistosoma mansoni*. Furthermore, DC treatment with certain immune modulating agents can influence the expression of co-stimulatory and co-inhibitory molecules. VitD-treated DCs displayed induced expression of PD-L1 and ILT3, concurrent with inhibited expression of CD80 and CD86. On the other hand, DCs under the influence of IL-10 had normal expression levels of CD80 and CD86 but over-expressed ILT3 and ILT4. It is also evident that DCs are strongly influenced by cues derived from the local environment. The well-documented effect of VitD, the major component of local skin milieu, is a clear example. The influence of other known tissue-related environmental factors on co-stimulation requires further elucidation. Thus, optimal understanding of the role of DC-derived signal II requires determining the total repertoire of co-stimulatory and co-inhibitory molecules expressed by different DC subsets and under different conditions.

In addition to the differential DC expression of co-stimulatory and co-inhibitory molecules, the respective ligands of these molecules are also described to be expressed by T cells following different kinetics. Some of these ligands are constitutively expressed, like CD28, whereas others are restricted to recently TCR-activated T cells such as 4-1BB and GITR. Furthermore, some of these ligands were shown to be exclusively expressed by certain types of effector T cells, like the Th1-specific expression of TIM3. Taken together, the different expression modalities of the co-stimulatory and co-inhibitory pathway constituents imply that these pathways are mobilized at certain stages of T cell priming and under specific conditions.

Despite the stimulatory or inhibitory nature of signal II, there are some indications pointing out a role in T cell polarization, typically undertaken by cytokine-based signal III. For instance, OX-40L/OX-40 and 4-1BBL/4-1BB pathways are proposed to promote the differentiation of Th2 and Th1 effector cells, respectively. Nevertheless, the observed polarizing effect was in many occasions revealed to be the mere outcome of promoted T cell survival rather than active polarization signaling mediated by these co-stimulatory or co-inhibitory molecules. Therefore, reported contributions of signal II to T cell differentiation should be interpreted carefully and further investigated.

The vast immunological consequences of signal II have transformed its pathways, both stimulatory and inhibitory, into therapeutic targets for the treatment of a wide variety of diseases. Mobilizing co-stimulatory pathways and blocking co-inhibitory interactions showed promising results in promoting anti-tumor immunity and it is proposed to be beneficial for the treatment of chronic viral responses. Assuming that mature DCs provide optimal positive co-stimulatory signals while priming anti-tumor T cells, blocking co-inhibitory pathways may augment the efficacy of these T cells. In that respect, concurrent targeting of multiple co-inhibitory pathways might be necessary. Neutralizing the key inhibitory check point CTLA-4 permits extensive primary T cell activation, but by itself is not sufficient for driving an anti-tumor immune response, especially in the case of advanced tumors. However, the additional circumvention of yet another co-inhibitory check point, which is dictated by the tumor itself, may solve this problem. Selecting the second inhibitory target would highly depend on the type of the treated tumor, as different types of tumors were revealed to preferentially express certain co-inhibitory receptors (PD-L1, PD-L2, B7-H3, etc.). The synergistic effects of such a combinatorial blocking strategy may not only mount efficient anti-tumor T cell responses, but also allow the persistence of such responses within the local tumor environment.

On the other hand, promoting tolerance by blocking activation and mobilizing co-inhibitory pathways is a promising strategy for raising allograft tolerance. Similarly, immune suppressant agents were also revealed to manipulate these pathways in a comparable manner to induce tolerance. Nevertheless, these therapeutic modalities should be applied with great care to avoid any possible adverse effects like inducing susceptibility to infection or autoimmune reactions. Targeting these therapies to a specific pathway or a specific cellular compartment, like a certain DC subset, may be an option to bypass any possible complications.

## Conflict of Interest Statement

The authors declare that the research was conducted in the absence of any commercial or financial relationships that could be construed as a potential conflict of interest.

## References

[B1] Agata Y. Kawasaki A. Nishimura H. Ishida Y. Tsubata T. Yagita H. (1996). Expression of the PD-1 antigen on the surface of stimulated mouse T and B lymphocytes. *Int. Immunol.* 8 765–772 8671665 10.1093/intimm/8.5.765

[B2] Akiba H. Nakano H. Nishinaka S. Shindo M. Kobata T. Atsuta M. (1998). CD27, a member of the tumor necrosis factor receptor superfamily, activates NF-kappaB and stress-activated protein kinase/c-Jun N-terminal kinase via TRAF2, TRAF5, and NF-kappaB-inducing kinase. *J. Biol. Chem.* 273 13353–13358 9582383 10.1074/jbc.273.21.13353

[B3] Alegre M. L. Noel P. J. Eisfelder B. J. Chuang E. Clark M. R. Reiner S. L. (1996). Regulation of surface and intracellular expression of CTLA4 on mouse T cells. *J. Immunol.* 157 4762–4770 8943377

[B4] Anderson A. C. Anderson D. E. Bregoli L. Hastings W. D. Kassam N. Lei C. (2007). Promotion of tissue inflammation by the immune receptor Tim-3 expressed on innate immune cells. *Science* 318 1141–1143 18006747 10.1126/science.1148536

[B5] Ara G. Baher A. Storm N. Horan T. Baikalov C. Brisan E. (2003). Potent activity of soluble B7RP-1-Fc in therapy of murine tumors in syngeneic hosts. *Int. J. Cancer* 103 501–507 12478666 10.1002/ijc.10831

[B6] Aruffo A. Seed B. (1987). Molecular cloning of a CD28 cDNA by a high-efficiency COS cell expression system. *Proc. Natl. Acad. Sci. U.S.A.* 84 8573–8577 2825196 10.1073/pnas.84.23.8573 PMC299587

[B7] Azuma H. Chandraker A. Nadeau K. Hancock W. W. Carpenter C. B. Tilney N. L. (1996). Blockade of T-cell costimulation prevents development of experimental chronic renal allograft rejection. *Proc. Natl. Acad. Sci. U.S.A.* 93 12439–12444 8901600 10.1073/pnas.93.22.12439 PMC38010

[B8] Azuma M. Ito D. Yagita H. Okumura K. Phillips J. H. Lanier L. L. (1993). B70 antigen is a second ligand for CTLA-4 and CD28. *Nature* 366 76–79 7694153 10.1038/366076a0

[B9] Banchereau J. Briere F. Caux C. Davoust J. Lebecque S. Liu Y. J. (2000). Immunobiology of dendritic cells. *Annu. Rev. Immunol.* 18 767–811 10837075 10.1146/annurev.immunol.18.1.767

[B10] Banchereau J. Steinman R. M. (1998). Dendritic cells and the control of immunity. *Nature* 392 245–252 9521319 10.1038/32588

[B11] Barber D. L. Wherry E. J. Masopust D. Zhu B. Allison J. P. Sharpe A. H. (2006). Restoring function in exhausted CD8 T cells during chronic viral infection. *Nature* 439 682–687 16382236 10.1038/nature04444

[B12] Bianchi E. Denti S. Granata A. Bossi G. Geginat J. Villa A. (2000). Integrin LFA-1 interacts with the transcriptional co-activator JAB1 to modulate AP-1 activity. *Nature* 404 617–621 10766246 10.1038/35007098

[B13] Blank C. Brown I. Marks R. Nishimura H. Honjo T. Gajewski T. F. (2003). Absence of programmed death receptor 1 alters thymic development and enhances generation of CD4/CD8 double-negative TCR-transgenic T cells. *J. Immunol.* 171 4574–4581 14568931 10.4049/jimmunol.171.9.4574

[B14] Bleijs D. A. Geijtenbeek T. B. Figdor C. G. van K. Y. (2001). DC-SIGN and LFA-1: a battle for ligand 1. *Trends Immunol.* 22 457–463 11473836 10.1016/s1471-4906(01)01974-3

[B15] Boise L. H. Minn A. J. Noel P. J. June C. H. Accavitti M. A. Lindsten T. (1995). CD28 costimulation can promote T cell survival by enhancing the expression of Bcl-XL. *Immunity* 3 87–98 7621080 10.1016/1074-7613(95)90161-2

[B16] Bour-Jordan H. Grogan J. L. Tang Q. Auger J. A. Locksley R. M. Bluestone J. A. (2003). CTLA-4 regulates the requirement for cytokine-induced signals in T(H)2 lineage commitment. *Nat. Immunol.* 4 182–188 12524538 10.1038/ni884

[B17] Boussiotis V. A. Freeman G. J. Griffin J. D. Gray G. S. Gribben J. G. Nadler L. M. (1994). CD2 is involved in maintenance and reversal of human alloantigen-specific clonal anergy. *J. Exp. Med.* 180 1665–1673 7525835 10.1084/jem.180.5.1665 PMC2191726

[B18] Brenk M. Scheler M. Koch S. Neumann J. Takikawa O. Hacker G. (2009). Tryptophan deprivation induces inhibitory receptors ILT3 and ILT4 on dendritic cells favoring the induction of human CD4+CD25+ Foxp3+ T regulatory cells. *J. Immunol.* 183 145–154 19535644 10.4049/jimmunol.0803277

[B19] Brunet J. F. Denizot F. Luciani M. F. Roux-Dosseto M. Suzan M. Mattei M. G. (1987). A new member of the immunoglobulin superfamily – CTLA-4. *Nature* 328 267–270 3496540 10.1038/328267a0

[B20] Bukczynski J. Wen T. Ellefsen K. Gauldie J. Watts T. H. (2004). Costimulatory ligand 4-1BBL (CD137L) as an efficient adjuvant for human antiviral cytotoxic T cell responses. *Proc. Natl. Acad. Sci. U.S.A.* 101 1291–1296 14745033 10.1073/pnas.0306567101 PMC337046

[B21] Burmeister Y. Lischke T. Dahler A. C. Mages H. W. Lam K. P. Coyle A. J. (2008). ICOS controls the pool size of effector-memory and regulatory T cells. *J. Immunol.* 180 774–782 18178815 10.4049/jimmunol.180.2.774

[B22] Bussmann C. Xia J. Allam J. P. Maintz L. Bieber T. Novak N. (2010). Early markers for protective mechanisms during rush venom immunotherapy. *Allergy* 65 1558–1565 20584008 10.1111/j.1398-9995.2010.02430.x

[B23] Butte M. J. Keir M. E. Phamduy T. B. Sharpe A. H. Freeman G. J. (2007). Programmed death-1 ligand 1 interacts specifically with the B7-1 costimulatory molecule to inhibit T cell responses. *Immunity* 27 111–122 17629517 10.1016/j.immuni.2007.05.016 PMC2707944

[B24] Cai G. Anumanthan A. Brown J. A. Greenfield E. A. Zhu B. Freeman G. J. (2008). CD160 inhibits activation of human CD4+ T cells through interaction with herpesvirus entry mediator. *Nat. Immunol.* 9 176–185 18193050 10.1038/ni1554

[B25] Caux C. Massacrier C. Vanbervliet B. Dubois B. Van K. C. Durand I. (1994). Activation of human dendritic cells through CD40 cross-linking. *J. Exp. Med.* 180 1263–1272 7523569 10.1084/jem.180.4.1263 PMC2191669

[B26] Cella M. Dohring C. Samaridis J. Dessing M. Brockhaus M. Lanzavecchia A. (1997). A novel inhibitory receptor (ILT3) expressed on monocytes, macrophages, and dendritic cells involved in antigen processing. *J. Exp. Med.* 185 1743–1751 9151699 10.1084/jem.185.10.1743 PMC2196312

[B27] Chang C. C. Ciubotariu R. Manavalan J. S. Yuan J. Colovai A. I. Piazza F. (2002). Tolerization of dendritic cells by T(S) cells: the crucial role of inhibitory receptors ILT3 and ILT4. *Nat. Immunol.* 3 237–243 11875462 10.1038/ni760

[B28] Chang C. C. Liu Z. Vlad G. Qin H. Qiao X. Mancini D. M. (2009). Ig-like transcript 3 regulates expression of proinflammatory cytokines and migration of activated T cells. *J. Immunol.* 182 5208–5216 19380766 10.4049/jimmunol.0804048

[B29] Chang C. C. Vlad G. D’Agati V. D. Liu Z. Zhang Q. Y. Witkowski P. (2010). BCL6 is required for differentiation of Ig-like transcript 3-Fc-induced CD8+ T suppressor cells. *J. Immunol.* 185 5714–5722 20935202 10.4049/jimmunol.1001732

[B30] Chapoval A. I. Ni J. Lau J. S. Wilcox R. A. Flies D. B. Liu D. (2001). B7-H3: a costimulatory molecule for T cell activation and IFN-gamma production. *Nat. Immunol.* 2 269–274 11224528 10.1038/85339

[B31] Chemnitz J. M. Parry R. V. Nichols K. E. June C. H. Riley J. L. (2004). SHP-1 and SHP-2 associate with immunoreceptor tyrosine-based switch motif of programmed death 1 upon primary human T cell stimulation, but only receptor ligation prevents T cell activation. *J. Immunol.* 173 945–954 15240681 10.4049/jimmunol.173.2.945

[B32] Cheung T. C. Steinberg M. W. Oborne L. M. Macauley M. G. Fukuyama S. Sanjo H. (2009). Unconventional ligand activation of herpesvirus entry mediator signals cell survival. *Proc. Natl. Acad. Sci. U.S.A.* 106 6244–6249 19332782 10.1073/pnas.0902115106 PMC2669392

[B33] Chiba S. Baghdadi M. Akiba H. Yoshiyama H. Kinoshita I. Dosaka-Akita H. (2012). Tumor-infiltrating DCs suppress nucleic acid-mediated innate immune responses through interactions between the receptor TIM-3 and the alarmin HMGB1. *Nat. Immunol.* 13 832–842 22842346 10.1038/ni.2376 PMC3622453

[B34] Choi I. H. Zhu G. Sica G. L. Strome S. E. Cheville J. C. Lau J. S. (2003). Genomic organization and expression analysis of B7-H4, an immune inhibitory molecule of the B7 family. *J. Immunol.* 171 4650–4654 14568939 10.4049/jimmunol.171.9.4650

[B35] Chu C. C. Ali N. Karagiannis P. Di M. P. Skowera A. Napolitano L. (2012). Resident CD141 (BDCA3)+ dendritic cells in human skin produce IL-10 and induce regulatory T cells that suppress skin inflammation. *J. Exp. Med.* 209 935–945 22547651 10.1084/jem.20112583 PMC3348099

[B36] Cohen A. D. Diab A. Perales M. A. Wolchok J. D. Rizzuto G. Merghoub T. (2006). Agonist anti-GITR antibody enhances vaccine-induced CD8(+) T-cell responses and tumor immunity. *Cancer Res.* 66 4904–4912 16651447 10.1158/0008-5472.CAN-05-2813 PMC2242844

[B37] Colonna M. Samaridis J. Cella M. Angman L. Allen R. L. O’Callaghan C. A. (1998). Human myelomonocytic cells express an inhibitory receptor for classical and nonclassical MHC class I molecules. *J. Immunol.* 160 3096–3100 9531263

[B38] Coquet J. M. Middendorp S. van der Horst G. Kind J. Veraar E. A. Xiao Y. (2012). The CD27 and CD70 costimulatory pathway inhibits effector function of T helper 17 cells and attenuates associated autoimmunity. *Immunity* 38 53–65 23159439 10.1016/j.immuni.2012.09.009

[B39] Couderc B. Zitvogel L. Douin-Echinard V. Djennane L. Tahara H. Favre G. (1998). Enhancement of antitumor immunity by expression of CD70 (CD27 ligand) or CD154 (CD40 ligand) costimulatory molecules in tumor cells. *Cancer Gene Ther.* 5 163–175 9622100

[B40] Coyle A. J. Lehar S. Lloyd C. Tian J. Delaney T. Manning S. (2000). The CD28-related molecule ICOS is required for effective T cell-dependent immune responses. *Immunity* 13 95–105 10933398 10.1016/s1074-7613(00)00011-x

[B41] Curiel T. J. Wei S. Dong H. Alvarez X. Cheng P. Mottram P. (2003). Blockade of B7-H1 improves myeloid dendritic cell-mediated antitumor immunity. *Nat. Med.* 9 562–567 12704383 10.1038/nm863

[B42] Davis S. J van der Merwe P. A. (1996). The structure and ligand interactions of CD2: implications for T-cell function. *Immunol. Today* 17 177–187 8871350 10.1016/0167-5699(96)80617-7

[B43] De Jong E. C. Smits H. H. Kapsenberg M. L. (2005). Dendritic cell-mediated T cell polarization. *Springer Semin. Immunopathol.* 26 289–307 15609003 10.1007/s00281-004-0167-1

[B44] Deppong C. Juehne T. I. Hurchla M. Friend L. D. Shah D. D. Rose C. M. (2006). Cutting edge: B and T lymphocyte attenuator and programmed death receptor-1 inhibitory receptors are required for termination of acute allergic airway inflammation. *J. Immunol.* 176 3909–3913 16547224 10.4049/jimmunol.176.7.3909

[B45] Derre L. Rivals J. P. Jandus C. Pastor S. Rimoldi D. Romero P. (2010). BTLA mediates inhibition of human tumor-specific CD8+ T cells that can be partially reversed by vaccination. *J. Clin. Invest.* 120 157–167 20038811 10.1172/JCI40070 PMC2799219

[B46] Dong H. Zhu G. Tamada K. Chen L. (1999). B7-H1, a third member of the B7 family, co-stimulates T-cell proliferation and interleukin-10 secretion. *Nat. Med.* 5 1365–1369 10581077 10.1038/70932

[B47] Driessens G. Kline J. Gajewski T. F. (2009). Costimulatory and coinhibitory receptors in anti-tumor immunity. *Immunol. Rev.* 229 126–144 19426219 10.1111/j.1600-065X.2009.00771.x PMC3278040

[B48] Elpek K. G. Yolcu E. S. Franke D. D. Lacelle C. Schabowsky R. H. Shirwan H. (2007). Ex vivo expansion of CD4+CD25+FoxP3+ T regulatory cells based on synergy between IL-2 and 4-1BB signaling. *J. Immunol.* 179 7295–7304 18025172 10.4049/jimmunol.179.11.7295

[B49] Esparza E. M. Arch R. H. (2005). Glucocorticoid-induced TNF receptor functions as a costimulatory receptor that promotes survival in early phases of T cell activation. *J. Immunol.* 174 7869–7874 15944292 10.4049/jimmunol.174.12.7869

[B50] Fallarino F. Ashikari A. Boon T. Gajewski T. F. (1997). Antigen-specific regression of established tumors induced by active immunization with irradiated IL-12- but not B7-1-transfected tumor cells. *Int. Immunol.* 9 1259–1269 9310829 10.1093/intimm/9.9.1259

[B51] Fallarino F. Grohmann U. Hwang K. W. Orabona C. Vacca C. Bianchi R. (2003). Modulation of tryptophan catabolism by regulatory T cells. *Nat. Immunol.* 4 1206–1212 14578884 10.1038/ni1003

[B52] Fife B. T. Pauken K. E. Eagar T. N. Obu T. Wu J. Tang Q. (2009). Interactions between PD-1 and PD-L1 promote tolerance by blocking the TCR-induced stop signal. *Nat. Immunol.* 10 1185–1192 19783989 10.1038/ni.1790 PMC2778301

[B53] Flynn S. Toellner K. M. Raykundalia C. Goodall M. Lane P. (1998). CD4 T cell cytokine differentiation: the B cell activation molecule, OX40 ligand, instructs CD4 T cells to express interleukin 4 and upregulates expression of the chemokine receptor, Blr-1. *J. Exp. Med.* 188 297–304 9670042 10.1084/jem.188.2.297 PMC2212448

[B54] Foell J. Strahotin S. O’Neil S. P. McCausland M. M. Suwyn C. Haber M. (2003). CD137 costimulatory T cell receptor engagement reverses acute disease in lupus-prone NZB × NZW F1 mice. *J. Clin. Invest.* 111 1505–1518 12750400 10.1172/JCI17662 PMC155050

[B55] Francisco L. M. Sage P. T. Sharpe A. H. (2010). The PD-1 pathway in tolerance and autoimmunity. *Immunol. Rev.* 236 219–242 20636820 10.1111/j.1600-065X.2010.00923.x PMC2919275

[B56] Francisco L. M. Salinas V. H. Brown K. E. Vanguri V. K. Freeman G. J. Kuchroo V. K. (2009). PD-L1 regulates the development, maintenance, and function of induced regulatory T cells. *J. Exp. Med.* 206 3015–3029 20008522 10.1084/jem.20090847 PMC2806460

[B57] Frauwirth K. A. Riley J. L. Harris M. H. Parry R. V. Rathmell J. C. Plas D. R. (2002). The CD28 signaling pathway regulates glucose metabolism. *Immunity* 16 769–777 12121659 10.1016/s1074-7613(02)00323-0

[B58] Freeman G. J. Freedman A. S. Segil J. M. Lee G. Whitman J. F. Nadler L. M. (1989). B7, a new member of the Ig superfamily with unique expression on activated and neoplastic B cells. *J. Immunol.* 143 2714–2722 2794510

[B59] French R. R. Chan H. T. Tutt A. L. Glennie M. J. (1999). CD40 antibody evokes a cytotoxic T-cell response that eradicates lymphoma and bypasses T-cell help. *Nat. Med.* 5 548–553 10229232 10.1038/8426

[B60] Green J. M. Noel P. J. Sperling A. I. Walunas T. L. Gray G. S. Bluestone J. A. (1994). Absence of B7-dependent responses in CD28-deficient mice. *Immunity* 1 501–508 7534617 10.1016/1074-7613(94)90092-2

[B61] Greenwald R. J. Freeman G. J. Sharpe A. H. (2005). The B7 family revisited. *Annu. Rev. Immunol.* 23 515–548 15771580 10.1146/annurev.immunol.23.021704.115611

[B62] Gregori S. Tomasoni D. Pacciani V. Scirpoli M. Battaglia M. Magnani C. F. (2010). Differentiation of type 1 T regulatory cells (Tr1) by tolerogenic DC-10 requires the IL-10-dependent ILT4/HLA-G pathway. *Blood* 116 935–944 20448110 10.1182/blood-2009-07-234872

[B63] Guo L. Li X. K. Funeshima N. Fujino M. Nagata Y. Kimura H. (2002). Prolonged survival in rat liver transplantation with mouse monoclonal antibody against an inducible costimulator (ICOS). *Transplantation* 73 1027–1032 11965027 10.1097/00007890-200204150-00003

[B64] Gurney A. L. Marsters S. A. Huang R. M. Pitti R. M. Mark D. T. Baldwin D. T. (1999). Identification of a new member of the tumor necrosis factor family and its receptor, a human ortholog of mouse GITR. *Curr. Biol.* 9 215–218 10074428 10.1016/s0960-9822(99)80093-1

[B65] Han B. K. White A. M. Dao K. H. Karp D. R. Wakeland E. K. Davis L. S. (2005). Increased prevalence of activated CD70+CD4+ T cells in the periphery of patients with systemic lupus erythematosus. *Lupus* 14 598–606 16175931 10.1191/0961203305lu2171oa

[B66] Hancock W. W. Buelow R. Sayegh M. H. Turka L. A. (1998). Antibody-induced transplant arteriosclerosis is prevented by graft expression of anti-oxidant and anti-apoptotic genes. *Nat. Med.* 4 1392–1396 9846576 10.1038/3982

[B67] Harrop J. A. McDonnell P. C. Brigham-Burke M. Lyn S. D. Minton J. Tan K. B. (1998). Herpesvirus entry mediator ligand (HVEM-L), a novel ligand for HVEM/TR2, stimulates proliferation of T cells and inhibits HT29 cell growth. *J. Biol. Chem.* 273 27548–27556 9765287 10.1074/jbc.273.42.27548

[B68] Hashiguchi M. Kobori H. Ritprajak P. Kamimura Y. Kozono H. Azuma M. (2008). Triggering receptor expressed on myeloid cell-like transcript 2 (TLT-2) is a counter-receptor for B7-H3 and enhances T cell responses. *Proc. Natl. Acad. Sci. U.S.A.* 105 10495–10500 18650384 10.1073/pnas.0802423105 PMC2492502

[B69] Hendriks J. Gravestein L. A. Tesselaar K. van Lier R. A. Schumacher T. N. Borst J. (2000). CD27 is required for generation and long-term maintenance of T cell immunity. *Nat. Immunol.* 1 433–440 11062504 10.1038/80877

[B70] Hendriks J. Xiao Y. Borst J. (2003). CD27 promotes survival of activated T cells and complements CD28 in generation and establishment of the effector T cell pool. *J. Exp. Med.* 198 1369–1380 14581610 10.1084/jem.20030916 PMC2194245

[B71] Hodi F. S. Mihm M. C. Soiffer R. J. Haluska F. G. Butler M. Seiden M. V. (2003). Biologic activity of cytotoxic T lymphocyte-associated antigen 4 antibody blockade in previously vaccinated metastatic melanoma and ovarian carcinoma patients. *Proc. Natl. Acad. Sci. U.S.A.* 100 4712–4717 12682289 10.1073/pnas.0830997100 PMC153621

[B72] Hofmeyer K. A. Ray A. Zang X. (2008). The contrasting role of B7-H3 1. *Proc. Natl. Acad. Sci. U.S.A.* 105 10277–10278 18650376 10.1073/pnas.0805458105 PMC2492485

[B73] Hunt J. S. Petroff M. G. McIntire R. H. Ober C. (2005). HLA-G and immune tolerance in pregnancy. *FASEB J.* 19 681–693 15857883 10.1096/fj.04-2078rev

[B74] Hurchla M. A. Sedy J. R. Gavrieli M. Drake C. G. Murphy T. L Murphy K. M. (2005). B and T lymphocyte attenuator exhibits structural and expression polymorphisms and is highly Induced in anergic CD4+ T cells. *J. Immunol.* 174 3377–3385 15749870 10.4049/jimmunol.174.6.3377

[B75] Hutloff A. Dittrich A. M. Beier K. C. Eljaschewitsch B. Kraft R. Anagnostopoulos I. (1999). ICOS is an inducible T-cell co-stimulator structurally and functionally related to CD28. *Nature* 397 263–266 9930702 10.1038/16717

[B76] Ito T. Wang Y. H. Duramad O. Hori T. Delespesse G. J. Watanabe N. (2005). TSLP-activated dendritic cells induce an inflammatory T helper type 2 cell response through OX40 ligand. *J. Exp. Med.* 202 1213–1223 16275760 10.1084/jem.20051135 PMC2213234

[B77] Jenkins M. K. Schwartz R. H. (1987). Antigen presentation by chemically modified splenocytes induces antigen-specific T cell unresponsiveness in vitro and in vivo. *J. Exp. Med.* 165 302–319 3029267 10.1084/jem.165.2.302 PMC2188516

[B78] Jenkins S. J. Perona-Wright G. Worsley A. G. Ishii N. MacDonald A. S. (2007). Dendritic cell expression of OX40 ligand acts as a costimulatory, not polarizing, signal for optimal Th2 priming and memory induction in vivo. *J. Immunol.* 179 3515–3523 17785785 10.4049/jimmunol.179.6.3515

[B79] Jensen M. A. Patterson K. C. Kumar A. A. Kumabe M. Franek B. S. Niewold T. B. (2012). Functional genetic polymorphisms in ILT3 are associated with decreased surface expression on dendritic cells and increased serum cytokines in lupus patients. *Ann. Rheum. Dis.* 10.1136/annrheumdis-2012-202024 [Epub ahead of print]. PMC3910490 22904259

[B80] Jensen M. A. Yanowitch R. N. Reder A. T. White D. M. Arnason B. G. (2010). Immunoglobulin-like transcript 3, an inhibitor of T cell activation, is reduced on blood monocytes during multiple sclerosis relapses and is induced by interferon beta-1b. *Mult. Scler.* 16 30–38 20007427 10.1177/1352458509352794

[B81] Jiang J. Zhu Y. Wu C. Shen Y. Wei W. Chen L. (2010). Tumor expression of B7-H4 predicts poor survival of patients suffering from gastric cancer. *Cancer Immunol. Immunother.* 59 1707–1714 20725832 10.1007/s00262-010-0900-7 PMC11031090

[B82] Jones N. D. Van Maurik A. Hara M. Spriewald B. M. Witzke O. Morris P. J. (2000). CD40–CD40 ligand-independent activation of CD8+ T cells can trigger allograft rejection. *J. Immunol.* 165 1111–1118 10878390 10.4049/jimmunol.165.2.1111

[B83] Ju X. S. Hacker C. Scherer B. Redecke V. Berger T. Schuler G. (2004). Immunoglobulin-like transcripts ILT2, ILT3 and ILT7 are expressed by human dendritic cells and down-regulated following activation. *Gene* 331 159–164 15094202 10.1016/j.gene.2004.02.018

[B84] Keir M. E. Liang S. C. Guleria I. Latchman Y. E. Qipo A. Albacker L. A. (2006). Tissue expression of PD-L1 mediates peripheral T cell tolerance. *J. Exp. Med.* 203 883–895 16606670 10.1084/jem.20051776 PMC2118286

[B85] Keller A. M. Schildknecht A. Xiao Y. van den Broek M. Borst J. (2008). Expression of costimulatory ligand CD70 on steady-state dendritic cells breaks CD8+ T cell tolerance and permits effective immunity. *Immunity* 29 934–946 19062317 10.1016/j.immuni.2008.10.009

[B86] Khong A. Nelson D. J. Nowak A. K. Lake R. A. Robinson B. W. (2012). The use of agonistic anti-CD40 therapy in treatments for cancer. *Int. Rev. Immunol.* 31 246–266 22804570 10.3109/08830185.2012.698338

[B87] Kim Y. J. Kim S. H. Mantel P. Kwon B. S. (1998). Human 4-1BB regulates CD28 co-stimulation to promote Th1 cell responses. *Eur. J. Immunol.* 28 881–890 9541583 10.1002/(SICI)1521-4141(199803)28:03<881::AID-IMMU881>3.0.CO;2-0

[B88] Kim-Schulze S. Scotto L. Vlad G. Piazza F. Lin H. Liu Z. (2006). Recombinant Ig-like transcript 3-Fc modulates T cell responses via induction of Th anergy and differentiation of CD8+ T suppressor cells. *J. Immunol.* 176 2790–2798 16493035 10.4049/jimmunol.176.5.2790

[B89] Kirkwood J. M. Butterfield L. H. Tarhini A. A. Zarour H. Kalinski P. Ferrone S. (2012). Immunotherapy of cancer in 2012. *CA Cancer J. Clin.* 62 309–335 22576456 10.3322/caac.20132 PMC3445708

[B90] Ko K. Yamazaki S. Nakamura K. Nishioka T. Hirota K. Yamaguchi T. (2005). Treatment of advanced tumors with agonistic anti-GITR mAb and its effects on tumor-infiltrating Foxp3+CD25+CD4+ regulatory T cells. *J. Exp. Med.* 202 885–891 16186187 10.1084/jem.20050940 PMC2213162

[B91] Kojima R. Kajikawa M. Shiroishi M. Kuroki K. Maenaka K. (2011). Molecular basis for herpesvirus entry mediator recognition by the human immune inhibitory receptor CD160 and its relationship to the cosignaling molecules BTLA and LIGHT. *J. Mol. Biol.* 413 762–772 21959263 10.1016/j.jmb.2011.09.018

[B92] Kroner A. Mehling M. Hemmer B. Rieckmann P. Toyka K. V. Maurer M. (2005). A PD-1 polymorphism is associated with disease progression in multiple sclerosis. *Ann. Neurol.* 58 50–57 15912506 10.1002/ana.20514

[B93] Kryczek I. Wei S. Zou L. Zhu G. Mottram P. Xu H. (2006a). Cutting edge: induction of B7-H4 on APCs through IL-10: novel suppressive mode for regulatory T cells. *J. Immunol.* 177 40–44 16785496 10.4049/jimmunol.177.1.40

[B94] Kryczek I. Zou L. Rodriguez P. Zhu G. Wei S. Mottram P. (2006b). B7-H4 expression identifies a novel suppressive macrophage population in human ovarian carcinoma. *J. Exp. Med.* 203 871–881 16606666 10.1084/jem.20050930 PMC2118300

[B95] Kuchroo V. K. Dardalhon V. Xiao S. Anderson A. C. (2008). New roles for TIM family members in immune regulation. *Nat. Rev. Immunol.* 8 577–580 18617884 10.1038/nri2366

[B96] La S. Kim J. Kwon B. S. Kwon B. (2002). Herpes simplex virus type 1 glycoprotein D inhibits T-cell proliferation. *Mol. Cells* 14 398–403 12521303

[B97] Lafferty K. J. Woolnough J. (1977). The origin and mechanism of the allograft reaction. *Immunol. Rev.* 35 231–262 330389 10.1111/j.1600-065x.1977.tb00241.x

[B98] Larsen C. P. Elwood E. T. Alexander D. Z. Ritchie S. C. Hendrix R. Tucker-Burden C. (1996). Long-term acceptance of skin and cardiac allografts after blocking CD40 and CD28 pathways. *Nature* 381 434–438 8632801 10.1038/381434a0

[B99] Latchman Y. Wood C. R. Chernova T. Chaudhary D. Borde M. Chernova I. (2001). PD-L2 is a second ligand for PD-1 and inhibits T cell activation. *Nat. Immunol.* 2 261–268 11224527 10.1038/85330

[B100] Leach D. R. Krummel M. F. Allison J. P. (1996). Enhancement of antitumor immunity by CTLA-4 blockade. *Science* 271 1734–1736 8596936 10.1126/science.271.5256.1734

[B101] Lee H. W. Park S. J. Choi B. K. Kim H. H. Nam K. O. Kwon B. S. (2002). 4-1BB promotes the survival of CD8+ T lymphocytes by increasing expression of Bcl-xL and Bfl-1. *J. Immunol.* 169 4882–4888 12391199 10.4049/jimmunol.169.9.4882

[B102] Lee W. W. Yang Z. Z. Li G. Weyand C. M. Goronzy J. J. (2007). Unchecked CD70 expression on T cells lowers threshold for T cell activation in rheumatoid arthritis. *J. Immunol.* 179 2609–2615 17675524 10.4049/jimmunol.179.4.2609 PMC2832914

[B103] Leitner J. Klauser C. Pickl W. F. Stockl J. Majdic O. Bardet A. F. (2009). B7-H3 is a potent inhibitor of human T-cell activation: no evidence for B7-H3 and TREML2 interaction. *Eur. J. Immunol.* 39 1754–1764 19544488 10.1002/eji.200839028 PMC2978551

[B104] Lesterhuis W. J. Punt C. J. Hato S. V. Eleveld-Trancikova D. Jansen B. J. Nierkens S. (2011). Platinum-based drugs disrupt STAT6-mediated suppression of immune responses against cancer in humans and mice. *J. Clin. Invest.* 121 3100–3108 21765211 10.1172/JCI43656 PMC3148725

[B105] Li Y. Zheng X. X. Li X. C. Zand M. S. Strom T. B. (1998). Combined costimulation blockade plus rapamycin but not cyclosporine produces permanent engraftment. *Transplantation* 66 1387–1388 9846527 10.1097/00007890-199811270-00021

[B106] Liang S. Horuzsko A. (2003). Mobilizing dendritic cells for tolerance by engagement of immune inhibitory receptors for HLA-G. *Hum. Immunol.* 64 1025–1032 14602231 10.1016/j.humimm.2003.08.348

[B107] Lichterfeld M. Kavanagh D. G. Williams K. L. Moza B. Mui S. K. Miura T. (2007). A viral CTL escape mutation leading to immunoglobulin-like transcript 4-mediated functional inhibition of myelomonocytic cells. *J. Exp. Med.* 204 2813–2824 18025130 10.1084/jem.20061865 PMC2118510

[B108] Lin S. C. Kuo C. C. Chan C. H. (2006). Association of a BTLA gene polymorphism with the risk of rheumatoid arthritis. *J. Biomed. Sci.* 13 853–860 17024343 10.1007/s11373-006-9113-7

[B109] Ling V. Wu P. W. Finnerty H. F. Bean K. M. Spaulding V. Fouser L. A. (2000). Cutting edge: identification of GL50, a novel B7-like protein that functionally binds to ICOS receptor. *J. Immunol.* 164 1653–1657 10657606 10.4049/jimmunol.164.4.1653

[B110] Linsley P. S. Brady W. Grosmaire L. Aruffo A. Damle N. K. Ledbetter J. A. (1991a). Binding of the B cell activation antigen B7 to CD28 costimulates T cell proliferation and interleukin 2 mRNA accumulation. *J. Exp. Med.* 173 721–730 1847722 10.1084/jem.173.3.721 PMC2118836

[B111] Linsley P. S. Brady W. Urnes M. Grosmaire L. S. Damle N. K. Ledbetter J. A. (1991b). CTLA-4 is a second receptor for the B cell activation antigen B7. *J. Exp. Med.* 174 561–569 1714933 10.1084/jem.174.3.561 PMC2118936

[B112] Linsley P. S. Clark E. A. Ledbetter J. A. (1990). T-cell antigen CD28 mediates adhesion with B cells by interacting with activation antigen B7/BB-1. *Proc. Natl. Acad. Sci. U.S.A.* 87 5031–5035 2164219 10.1073/pnas.87.13.5031 PMC54255

[B113] Liu X. Bai X. F. Wen J. Gao J. X. Liu J. Lu P. (2001). B7H costimulates clonal expansion of, and cognate destruction of tumor cells by, CD8(+) T lymphocytes in vivo. *J. Exp. Med.* 194 1339–1348 11696598 10.1084/jem.194.9.1339 PMC2195972

[B114] Lohning M. Hutloff A. Kallinich T. Mages H. W. Bonhagen K. Radbruch A. (2003). Expression of ICOS in vivo defines CD4+ effector T cells with high inflammatory potential and a strong bias for secretion of interleukin 10. *J. Exp. Med.* 197 181–193 12538658 10.1084/jem.20020632 PMC2193816

[B115] Lorenz M. G. Kantor J. A. Schlom J. Hodge J. W. (1999). Anti-tumor immunity elicited by a recombinant vaccinia virus expressing CD70 (CD27L). *Hum. Gene Ther.* 10 1095–1103 10340542 10.1089/10430349950018094

[B116] Magistrelli G. Jeannin P. Herbault N. Benoit De C. A. Gauchat J. F. Bonnefoy J. Y. (1999). A soluble form of CTLA-4 generated by alternative splicing is expressed by nonstimulated human T cells. *Eur. J. Immunol.* 29 3596–3602 10556814 10.1002/(SICI)1521-4141(199911)29:11<3596::AID-IMMU3596>3.0.CO;2-Y

[B117] Manavalan J. S. Rossi P. C. Vlad G. Piazza F. Yarilina A. Cortesini R. (2003). High expression of ILT3 and ILT4 is a general feature of tolerogenic dendritic cells. *Transpl. Immunol.* 11 245–258 12967778 10.1016/S0966-3274(03)00058-3

[B118] Manocha M. Rietdijk S. Laouar A. Liao G. Bhan A. Borst J. (2009). Blocking CD27-CD70 costimulatory pathway suppresses experimental colitis. *J. Immunol.* 183 270–276 19525396 10.4049/jimmunol.0802424 PMC4232384

[B119] Martin E. O’Sullivan B. Low P. Thomas R. (2003). Antigen-specific suppression of a primed immune response by dendritic cells mediated by regulatory T cells secreting interleukin-10. *Immunity* 18 155–167 12530984 10.1016/s1074-7613(02)00503-4

[B120] Martin-Orozco N. Li Y. Wang Y. Liu S. Hwu P. Liu Y. J. (2010). Melanoma cells express ICOS ligand to promote the activation and expansion of T-regulatory cells. *Cancer Res.* 70 9581–9590 21098714 10.1158/0008-5472.CAN-10-1379 PMC3058814

[B121] Matzinger P. (2002). The danger model: a renewed sense of self. *Science* 296 301–305 11951032 10.1126/science.1071059

[B122] McAdam A. J. Chang T. T. Lumelsky A. E. Greenfield E. A. Boussiotis V. A. Duke-Cohan J. S. (2000). Mouse inducible costimulatory molecule (ICOS) expression is enhanced by CD28 costimulation and regulates differentiation of CD4+ T cells. *J. Immunol.* 165 5035–5040 11046032 10.4049/jimmunol.165.9.5035

[B123] Medzhitov R. Janeway C. A. Jr. (2002). Decoding the patterns of self and nonself by the innate immune system. *Science* 296 298–300 11951031 10.1126/science.1068883

[B124] Meyers J. H. Chakravarti S. Schlesinger D. Illes Z. Waldner H. Umetsu S. E. (2005). TIM-4 is the ligand for TIM-1, and the TIM-1-TIM-4 interaction regulates T cell proliferation. *Nat. Immunol.* 6 455–464 15793576 10.1038/ni1185

[B125] Miga A. J. Masters S. R. Durell B. G. Gonzalez M. Jenkins M. K. Maliszewski C. (2001). Dendritic cell longevity and T cell persistence is controlled by CD154–CD40 interactions. *Eur. J. Immunol.* 31 959–965 11241301 10.1002/1521-4141(200103)31:3<959::aid-immu959>3.0.co;2-a

[B126] Miyanishi M. Tada K. Koike M. Uchiyama Y. Kitamura T. Nagata S. (2007). Identification of Tim4 as a phosphatidylserine receptor. *Nature* 450 435–439 17960135 10.1038/nature06307

[B127] Mizui M. Shikina T. Arase H. Suzuki K. Yasui T. Rennert P. D. (2008). Bimodal regulation of T cell-mediated immune responses by TIM-4. *Int. Immunol.* 20 695–708 18367551 10.1093/intimm/dxn029

[B128] Monney L. Sabatos C. A. Gaglia J. L. Ryu A. Waldner H. Chernova T. (2002). Th1-specific cell surface protein Tim-3 regulates macrophage activation and severity of an autoimmune disease. *Nature* 415 536–541 11823861 10.1038/415536a

[B129] Montgomery R. I. Warner M. S. Lum B. J. Spear P. G. (1996). Herpes simplex virus-1 entry into cells mediated by a novel member of the TNF/NGF receptor family. *Cell* 87 427–436 8898196 10.1016/s0092-8674(00)81363-x

[B130] Morel Y. Schiano de Colella J. M. Harrop J. Deen K. C. Holmes S. D. Wattam T. A. (2000). Reciprocal expression of the TNF family receptor herpes virus entry mediator and its ligand LIGHT on activated T cells: LIGHT down-regulates its own receptor. *J. Immunol.* 165 4397–4404 11035077 10.4049/jimmunol.165.8.4397

[B131] Morel Y. Truneh A. Sweet R. W. Olive D. Costello R. T. (2001). The TNF superfamily members LIGHT and CD154 (CD40 ligand) costimulate induction of dendritic cell maturation and elicit specific CTL activity. *J. Immunol.* 167 2479–2486 11509586 10.4049/jimmunol.167.5.2479

[B132] Morita M. Fujino M. Jiang G. Kitazawa Y. Xie L. Azuma M. (2010). PD-1/B7-H1 interaction contribute to the spontaneous acceptance of mouse liver allograft. *Am. J. Transplant.* 10 40–46 19889124 10.1111/j.1600-6143.2009.02859.x PMC2887673

[B133] Nakayama M. Akiba H. Takeda K. Kojima Y. Hashiguchi M. Azuma M. (2009). Tim-3 mediates phagocytosis of apoptotic cells and cross-presentation. *Blood* 113 3821–3830 19224762 10.1182/blood-2008-10-185884

[B134] Ndejembi M. P. Teijaro J. R. Patke D. S. Bingaman A. W. Chandok M. R. Azimzadeh A. (2006). Control of memory CD4 T cell recall by the CD28/B7 costimulatory pathway. *J. Immunol.* 177 7698–7706 17114440 10.4049/jimmunol.177.11.7698

[B135] Nielsen C. Hansen D. Husby S. Jacobsen B. B. Lillevang S. T. (2003). Association of a putative regulatory polymorphism in the PD-1 gene with susceptibility to type 1 diabetes. *Tissue Antigens* 62 492–497 14617032 10.1046/j.1399-0039.2003.00136.x

[B136] Nishimura H. Honjo T. Minato N. (2000). Facilitation of beta selection and modification of positive selection in the thymus of PD-1-deficient mice. *J. Exp. Med.* 191 891–898 10704469 10.1084/jem.191.5.891 PMC2195853

[B137] Nishimura H. Nose M. Hiai H. Minato N. Honjo T. (1999). Development of lupus-like autoimmune diseases by disruption of the PD-1 gene encoding an ITIM motif-carrying immunoreceptor. *Immunity* 11 141–151 10485649 10.1016/s1074-7613(00)80089-8

[B138] Nishimura H. Okazaki T. Tanaka Y. Nakatani K. Hara M. Matsumori A. (2001). Autoimmune dilated cardiomyopathy in PD-1 receptor-deficient mice. *Science* 291 319–322 11209085 10.1126/science.291.5502.319

[B139] Nocentini G. Giunchi L. Ronchetti S. Krausz L. T. Bartoli A. Moraca R. (1997). A new member of the tumor necrosis factor/nerve growth factor receptor family inhibits T cell receptor-induced apoptosis. *Proc. Natl. Acad. Sci. U.S.A.* 94 6216–6221 9177197 10.1073/pnas.94.12.6216 PMC21029

[B140] Oflazoglu E. Boursalian T. E. Zeng W. Edwards A. C. Duniho S. McEarchern J. A. (2009). Blocking of CD27-CD70 pathway by anti-CD70 antibody ameliorates joint disease in murine collagen-induced arthritis. *J. Immunol.* 183 3770–3777 19710474 10.4049/jimmunol.0901637

[B141] Ohshima Y. Tanaka Y. Tozawa H. Takahashi Y. Maliszewski C. Delespesse G. (1997). Expression and function of OX40 ligand on human dendritic cells. *J. Immunol.* 159 3838–3848 9378971

[B142] Oki M. Watanabe N. Owada T. Oya Y. Ikeda K. Saito Y. (2011). A functional polymorphism in B and T lymphocyte attenuator is associated with susceptibility to rheumatoid arthritis. *Clin. Dev. Immunol.* 2011 305656 10.1155/2011/305656 PMC3049324 21403914

[B143] Oya Y. Watanabe N. Owada T. Oki M. Hirose K. Suto A. (2008). Development of autoimmune hepatitis-like disease and production of autoantibodies to nuclear antigens in mice lacking B and T lymphocyte attenuator. *Arthritis Rheum.* 58 2498–2510 18668554 10.1002/art.23674 PMC2782777

[B144] Ozkaynak E. Wang L. Goodearl A. McDonald K. Qin S. O’Keefe T. (2002). Programmed death-1 targeting can promote allograft survival. *J. Immunol.* 169 6546–6553 12444166 10.4049/jimmunol.169.11.6546

[B145] Park H. Li Z. Yang X. O. Chang S. H. Nurieva R. Wang Y. H. (2005). A distinct lineage of CD4 T cells regulates tissue inflammation by producing interleukin 17. *Nat. Immunol.* 6 1133–1141 16200068 10.1038/ni1261 PMC1618871

[B146] Paterson D. J. Jefferies W. A. Green J. R. Brandon M. R. Corthesy P. Puklavec M. (1987). Antigens of activated rat T lymphocytes including a molecule of 50,000 Mr detected only on CD4 positive T blasts. *Mol. Immunol.* 24 1281–1290 2828930 10.1016/0161-5890(87)90122-2

[B147] Patsoukis N. Brown J. Petkova V. Liu F. Li L. Boussiotis V. A. (2012). Selective effects of PD-1 on Akt and Ras pathways regulate molecular components of the cell cycle and inhibit T cell proliferation. *Sci. Signal.* 5 ra46 10.1126/scisignal.2002796 PMC5498435 22740686

[B148] Penna G. Roncari A. Amuchastegui S. Daniel K. C. Berti E. Colonna M. (2005). Expression of the inhibitory receptor ILT3 on dendritic cells is dispensable for induction of CD4+Foxp3+ regulatory T cells by 1,25-dihydroxyvitamin D3. *Blood* 106 3490–3497 16030186 10.1182/blood-2005-05-2044

[B149] Peperzak V. Xiao Y. Veraar E. A. Borst J. (2010). CD27 sustains survival of CTLs in virus-infected nonlymphoid tissue in mice by inducing autocrine IL-2 production. *J. Clin. Invest.* 120 168–178 19955658 10.1172/JCI40178 PMC2798690

[B150] Pollok K. E. Kim Y. J. Zhou Z. Hurtado J. Kim K. K. Pickard R. T. (1993). Inducible T cell antigen 4-1BB. Analysis of expression and function. *J. Immunol.* 150 771–781 7678621

[B151] Prasad D. V. Nguyen T. Li Z. Yang Y. Duong J. Wang Y. (2004). Murine B7-H3 is a negative regulator of T cells. *J. Immunol.* 173 2500–2506 15294965 10.4049/jimmunol.173.4.2500

[B152] Prasad D. V. Richards S. Mai X. M. Dong C. (2003). B7S1, a novel B7 family member that negatively regulates T cell activation. *Immunity* 18 863–873 12818166 10.1016/s1074-7613(03)00147-x

[B153] Probst H. C. McCoy K. Okazaki T. Honjo T van den Broek M. (2005). Resting dendritic cells induce peripheral CD8+ T cell tolerance through PD-1 and CTLA-4. *Nat. Immunol.* 6 280–286 15685176 10.1038/ni1165

[B154] Prokunina L. Castillejo-Lopez C. Oberg F. Gunnarsson I. Berg L. Magnusson V. (2002). A regulatory polymorphism in PDCD1 is associated with susceptibility to systemic lupus erythematosus in humans. *Nat. Genet.* 32 666–669 12402038 10.1038/ng1020

[B155] Purvis H. A. Stoop J. N. Mann J. Woods S. Kozijn A. E. Hambleton S. (2010). Low-strength T-cell activation promotes Th17 responses. *Blood* 116 4829–4837 20713963 10.1182/blood-2010-03-272153 PMC3223073

[B156] Quezada S. A. Jarvinen L. Z. Lind E. F. Noelle R. J. (2004). CD40/CD154 interactions at the interface of tolerance and immunity. *Annu. Rev. Immunol.* 22 307–328 15032580 10.1146/annurev.immunol.22.012703.104533

[B157] Qureshi O. S. Zheng Y. Nakamura K. Attridge K. Manzotti C. Schmidt E. M. (2011). Trans-endocytosis of CD80 and CD86: a molecular basis for the cell-extrinsic function of CTLA-4. *Science* 332 600–603 21474713 10.1126/science.1202947 PMC3198051

[B158] Read S. Malmstrom V. Powrie F. (2000). Cytotoxic T lymphocyte-associated antigen 4 plays an essential role in the function of CD25(+)CD4(+) regulatory cells that control intestinal inflammation. *J. Exp. Med.* 192 295–302 10899916 10.1084/jem.192.2.295 PMC2193261

[B159] Riley J. L. (2009). PD-1 signaling in primary T cells. *Immunol. Rev.* 229 114–125 19426218 10.1111/j.1600-065X.2009.00767.x PMC3424066

[B160] Rochat M. K. Ege M. J. Plabst D. Steinle J. Bitter S. Braun-Fahrlander C. (2010). Maternal vitamin D intake during pregnancy increases gene expression of ILT3 and ILT4 in cord blood. *Clin. Exp. Allergy* 40 786–794 20030662 10.1111/j.1365-2222.2009.03428.x

[B161] Rodriguez-Manzanet R. Meyers J. H. Balasubramanian S. Slavik J. Kassam N. Dardalhon V. (2008). TIM-4 expressed on APCs induces T cell expansion and survival. *J. Immunol.* 180 4706–4713 18354194 10.4049/jimmunol.180.7.4706 PMC2948965

[B162] Rogers P. R. Song J. Gramaglia I. Killeen N. Croft M. (2001). OX40 promotes Bcl-xL and Bcl-2 expression and is essential for long-term survival of CD4 T cells. *Immunity* 15 445–455 11567634 10.1016/s1074-7613(01)00191-1

[B163] Ronchetti S. Zollo O. Bruscoli S. Agostini M. Bianchini R. Nocentini G. (2004). GITR, a member of the TNF receptor superfamily, is costimulatory to mouse T lymphocyte subpopulations. *Eur. J. Immunol.* 34 613–622 14991590 10.1002/eji.200324804

[B164] Rothlein R. Dustin M. L. Marlin S. D. Springer T. A. (1986). A human intercellular adhesion molecule (ICAM-1) distinct from LFA-1. *J. Immunol.* 137 1270–1274 3525675

[B165] Rottman J. B. Smith T. Tonra J. R. Ganley K. Bloom T. Silva R. (2001). The costimulatory molecule ICOS plays an important role in the immunopathogenesis of EAE. *Nat. Immunol.* 2 605–611 11429544 10.1038/89750

[B166] Ruby C. E. Yates M. A. Hirschhorn-Cymerman D. Chlebeck P. Wolchok J. D. Houghton A. N. (2009). Cutting edge: OX40 agonists can drive regulatory T cell expansion if the cytokine milieu is right. *J. Immunol.* 183 4853–4857 19786544 10.4049/jimmunol.0901112 PMC4625917

[B167] Rudd C. E. Taylor A. Schneider H. (2009). CD28 and CTLA-4 coreceptor expression and signal transduction. *Immunol. Rev.* 229 12–26 19426212 10.1111/j.1600-065X.2009.00770.x PMC4186963

[B168] Rulifson I. C. Sperling A. I. Fields P. E. Fitch F. W. Bluestone J. A. (1997). CD28 costimulation promotes the production of Th2 cytokines. *J. Immunol.* 158 658–665 8992981

[B169] Salomon B. Bluestone J. A. (1998). LFA-1 interaction with ICAM-1 and ICAM-2 regulates Th2 cytokine production. *J. Immunol.* 161 5138–5142 9820482

[B170] Sanderson K. Scotland R. Lee P. Liu D. Groshen S. Snively J. (2005). Autoimmunity in a phase I trial of a fully human anti-cytotoxic T-lymphocyte antigen-4 monoclonal antibody with multiple melanoma peptides and Montanide ISA 51 for patients with resected stages III and IV melanoma. *J. Clin. Oncol.* 23 741–750 15613700 10.1200/JCO.2005.01.128

[B171] Saoulli K. Lee S. Y. Cannons J. L. Yeh W. C. Santana A. Goldstein M. D. (1998). CD28-independent, TRAF2-dependent costimulation of resting T cells by 4-1BB ligand. *J. Exp. Med.* 187 1849–1862 9607925 10.1084/jem.187.11.1849 PMC2212301

[B172] Schoenberger S. P. Toes R. E. van der Voort E. I. Offringa R. Melief C. J. (1998). T-cell help for cytotoxic T lymphocytes is mediated by CD40–CD40L interactions. *Nature* 393 480–483 9624005 10.1038/31002

[B173] Scholer A. Hugues S. Boissonnas A. Fetler L. Amigorena S. (2008). Intercellular adhesion molecule-1-dependent stable interactions between T cells and dendritic cells determine CD8+ T cell memory. *Immunity* 28 258–270 18275834 10.1016/j.immuni.2007.12.016

[B174] Schulz O. Edwards A. D. Schito M. Aliberti J. Manickasingham S. Sher A. (2000). CD40 triggering of heterodimeric IL-12 p70 production by dendritic cells in vivo requires a microbial priming signal. *Immunity* 13 453–462 11070164 10.1016/s1074-7613(00)00045-5

[B175] Schwartz R. H. (1997). T cell clonal anergy. *Curr. Opin. Immunol.* 9 351–357 9203408 10.1016/s0952-7915(97)80081-7

[B176] Sedy J. R. Gavrieli M. Potter K. G. Hurchla M. A. Lindsley R. C. Hildner K. (2005). B and T lymphocyte attenuator regulates T cell activation through interaction with herpesvirus entry mediator. *Nat. Immunol.* 6 90–98 15568026 10.1038/ni1144

[B177] Semnani R. T. Nutman T. B. Hochman P. Shaw S Van Seventer G. A. (1994). Costimulation by purified intercellular adhesion molecule 1 and lymphocyte function-associated antigen 3 induces distinct proliferation, cytokine and cell surface antigen profiles in human “naive” and “memory” CD4+ T cells. *J. Exp. Med.* 180 2125–2135 7525848 10.1084/jem.180.6.2125 PMC2191787

[B178] Seo S. K. Choi J. H. Kim Y. H. Kang W. J. Park H. Y. Suh J. H. (2004). 4-1BB-mediated immunotherapy of rheumatoid arthritis. *Nat. Med.* 10 1088–1094 15448685 10.1038/nm1107

[B179] Sharpe A. H. Wherry E. J. Ahmed R. Freeman G. J. (2007). The function of programmed cell death 1 and its ligands in regulating autoimmunity and infection. *Nat. Immunol.* 8 239–245 17304234 10.1038/ni1443

[B180] Shaw J. Wang Y. H. Ito T. Arima K. Liu Y. J. (2010). Plasmacytoid dendritic cells regulate B-cell growth and differentiation via CD70. *Blood* 115 3051–3057 20139096 10.1182/blood-2009-08-239145 PMC2858470

[B181] Shimizu J. Yamazaki S. Takahashi T. Ishida Y. Sakaguchi S. (2002). Stimulation of CD25(+)CD4(+) regulatory T cells through GITR breaks immunological self-tolerance. *Nat. Immunol.* 3 135–142 11812990 10.1038/ni759

[B182] Shrikant P. Khoruts A. Mescher M. F. (1999). CTLA-4 blockade reverses CD8+ T cell tolerance to tumor by a CD4+ T cell- and IL-2-dependent mechanism. *Immunity* 11 483–493 10549630 10.1016/s1074-7613(00)80123-5

[B183] Shuford W. W. Klussman K. Tritchler D. D. Loo D. T. Chalupny J. Siadak A. W. (1997). 4-1BB costimulatory signals preferentially induce CD8+ T cell proliferation and lead to the amplification in vivo of cytotoxic T cell responses. *J. Exp. Med.* 186 47–55 9206996 10.1084/jem.186.1.47 PMC2198949

[B184] Sica G. L. Choi I. H. Zhu G. Tamada K. Wang S. D. Tamura H. (2003). B7-H4, a molecule of the B7 family, negatively regulates T cell immunity 1. *Immunity* 18 849–861 12818165 10.1016/s1074-7613(03)00152-3

[B185] Sigmundsdottir H. Butcher E. C. (2008). Environmental cues, dendritic cells and the programming of tissue-selective lymphocyte trafficking. *Nat. Immunol.* 9 981–987 18711435 10.1038/ni.f.208 PMC3171274

[B186] So T. Croft M. (2007). Cutting edge: OX40 inhibits TGF-beta- and antigen-driven conversion of naive CD4 T cells into CD25+Foxp3+ T cells. *J. Immunol.* 179 1427–1430 17641007 10.4049/jimmunol.179.3.1427

[B187] So T. Song J. Sugie K. Altman A. Croft M. (2006). Signals from OX40 regulate nuclear factor of activated T cells c1 and T cell helper 2 lineage commitment. *Proc. Natl. Acad. Sci. U.S.A.* 103 3740–3745 16501042 10.1073/pnas.0600205103 PMC1450148

[B188] Soares H. Waechter H. Glaichenhaus N. Mougneau E. Yagita H. Mizenina O. (2007). A subset of dendritic cells induces CD4+ T cells to produce IFN-gamma by an IL-12-independent but CD70-dependent mechanism in vivo. *J. Exp. Med.* 204 1095–1106 17438065 10.1084/jem.20070176 PMC2118574

[B189] Song J. Lei F. T. Xiong X. Haque R. (2008). Intracellular signals of T cell costimulation. *Cell. Mol. Immunol.* 5 239–247 18761811 10.1038/cmi.2008.30 PMC4651295

[B190] Soroosh P. Ine S. Sugamura K. Ishii N. (2006). OX40–OX40 ligand interaction through T cell-T cell contact contributes to CD4 T cell longevity. *J. Immunol.* 176 5975–5987 16670306 10.4049/jimmunol.176.10.5975

[B191] Sotomayor E. M. Borrello I. Tubb E. Allison J. P. Levitsky H. I. (1999). In vivo blockade of CTLA-4 enhances the priming of responsive T cells but fails to prevent the induction of tumor antigen-specific tolerance. *Proc. Natl. Acad. Sci. U.S.A.* 96 11476–11481 10500201 10.1073/pnas.96.20.11476 PMC18058

[B192] Springer T. A. Davignon D. Ho M. K. Kurzinger K. Martz E. Sanchez-Madrid F. (1982). LFA-1 and Lyt-2,3, molecules associated with T lymphocyte-mediated killing; and Mac-1, an LFA-1 homologue associated with complement receptor function. *Immunol. Rev.* 68 171–195 6184305 10.1111/j.1600-065x.1982.tb01064.x

[B193] Steinman R. M. Cohn Z. A. (1973). Identification of a novel cell type in peripheral lymphoid organs of mice. I. Morphology, quantitation, tissue distribution. *J. Exp. Med.* 137 1142–1162 10.1084/jem.137.5.1142 PMC2139237 4573839

[B194] Steinman R. M. Nussenzweig M. C. (2002). Avoiding horror autotoxicus: the importance of dendritic cells in peripheral T cell tolerance. *Proc. Natl. Acad. Sci. U.S.A.* 99 351–358 11773639 10.1073/pnas.231606698 PMC117564

[B195] Stephens G. L. McHugh R. S. Whitters M. J. Young D. A. Luxenberg D. Carreno B. M. (2004). Engagement of glucocorticoid-induced TNFR family-related receptor on effector T cells by its ligand mediates resistance to suppression by CD4+CD25+ T cells. *J. Immunol.* 173 5008–5020 15470044 10.4049/jimmunol.173.8.5008

[B196] Strome S. E. Dong H. Tamura H. Voss S. G. Flies D. B. Tamada K. (2003). B7-H1 blockade augments adoptive T-cell immunotherapy for squamous cell carcinoma. *Cancer Res.* 63 6501–6505 14559843

[B197] Suciu-Foca N. Feirt N. Zhang Q. Y. Vlad G. Liu Z. Lin H. (2007). Soluble Ig-like transcript 3 inhibits tumor allograft rejection in humanized SCID mice and T cell responses in cancer patients. *J. Immunol.* 178 7432–7441 17513794 10.4049/jimmunol.178.11.7432

[B198] Sugamura K. Ishii N. Weinberg A. D. (2004). Therapeutic targeting of the effector T-cell co-stimulatory molecule OX40. *Nat. Rev. Immunol.* 4 420–431 15173831 10.1038/nri1371

[B199] Suh W. K. Gajewska B. U. Okada H. Gronski M. A. Bertram E. M. Dawicki W. (2003). The B7 family member B7-H3 preferentially down-regulates T helper type 1-mediated immune responses 1. *Nat. Immunol.* 4 899–906 12925852 10.1038/ni967

[B200] Suh W. K. Wang S. Duncan G. S. Miyazaki Y. Cates E. Walker T. (2006). Generation and characterization of B7-H4/B7S1/B7x-deficient mice. *Mol. Cell. Biol.* 26 6403–6411 16914726 10.1128/MCB.00755-06 PMC1592821

[B201] Sun Y. Lin X. Chen H. M. Wu Q. Subudhi S. K. Chen L. (2002). Administration of agonistic anti-4-1BB monoclonal antibody leads to the amelioration of experimental autoimmune encephalomyelitis. *J. Immunol.* 168 1457–1465 11801689 10.4049/jimmunol.168.3.1457

[B202] Sutmuller R. P. van Duivenvoorde L. M. Elsas van A. Schumacher T. N. Wildenberg M. E. Allison J. P. (2001). Synergism of cytotoxic T lymphocyte-associated antigen 4 blockade and depletion of CD25(+) regulatory T cells in antitumor therapy reveals alternative pathways for suppression of autoreactive cytotoxic T lymphocyte responses. *J. Exp. Med.* 194 823–832 11560997 10.1084/jem.194.6.823 PMC2195955

[B203] Tai X. Cowan M. Feigenbaum L. Singer A. (2005). CD28 costimulation of developing thymocytes induces Foxp3 expression and regulatory T cell differentiation independently of interleukin 2. *Nat. Immunol.* 6 152–162 15640801 10.1038/ni1160

[B204] Takeda I. Ine S. Killeen N. Ndhlovu L. C. Murata K. Satomi S. (2004). Distinct roles for the OX40–OX40 ligand interaction in regulatory and nonregulatory T cells. *J. Immunol.* 172 3580–3589 15004159 10.4049/jimmunol.172.6.3580

[B205] Tamada K. Shimozaki K. Chapoval A. I. Zhai Y. Su J. Chen S. F. (2000a). LIGHT, a TNF-like molecule, costimulates T cell proliferation and is required for dendritic cell-mediated allogeneic T cell response. *J. Immunol.* 164 4105–4110 10754304 10.4049/jimmunol.164.8.4105

[B206] Tamada K. Shimozaki K. Chapoval A. I. Zhu G. Sica G. Flies D. (2000b). Modulation of T-cell-mediated immunity in tumor and graft-versus-host disease models through the LIGHT co-stimulatory pathway. *Nat. Med.* 6 283–289 10700230 10.1038/73136

[B207] Tao R. Wang L. Murphy K. M. Fraser C. C. Hancock W. W. (2008). Regulatory T cell expression of herpesvirus entry mediator suppresses the function of B and T lymphocyte attenuator-positive effector T cells. *J. Immunol.* 180 6649–6655 18453584 10.4049/jimmunol.180.10.6649

[B208] Taylor P. A. Lees C. J. Fournier S. Allison J. P. Sharpe A. H. Blazar B. R. (2004). B7 expression on T cells down-regulates immune responses through CTLA-4 ligation via T–T interactions [corrections]. *J. Immunol.* 172 34–39 14688306 10.4049/jimmunol.172.1.34

[B209] Tesselaar K. Xiao Y. Arens R. van Schijndel G. M. Schuurhuis D. H. Mebius R. E. (2003). Expression of the murine CD27 ligand CD70 in vitro and in vivo. *J. Immunol.* 170 33–40 12496380 10.4049/jimmunol.170.1.33

[B210] Tivol E. A. Borriello F. Schweitzer A. N. Lynch W. P. Bluestone J. A. Sharpe A. H. (1995). Loss of CTLA-4 leads to massive lymphoproliferation and fatal multiorgan tissue destruction, revealing a critical negative regulatory role of CTLA-4. *Immunity* 3 541–547 7584144 10.1016/1074-7613(95)90125-6

[B211] Tone M. Tone Y. Adams E. Yates S. F. Frewin M. R. Cobbold S. P. (2003). Mouse glucocorticoid-induced tumor necrosis factor receptor ligand is costimulatory for T cells. *Proc. Natl. Acad. Sci. U.S.A.* 100 15059–15064 14608036 10.1073/pnas.2334901100 PMC299905

[B212] Townsend S. E. Allison J. P. (1993). Tumor rejection after direct costimulation of CD8+ T cells by B7-transfected melanoma cells. *Science* 259 368–370 7678351 10.1126/science.7678351

[B213] Unger W. W. Laban S. Kleijwegt F. S. van der Slik A. R. Roep B. O. (2009). Induction of Treg by monocyte-derived DC modulated by vitamin D3 or dexamethasone: differential role for PD-L1. *Eur. J. Immunol.* 39 3147–3159 19688742 10.1002/eji.200839103

[B214] Uraushihara K. Kanai T. Ko K. Totsuka T. Makita S. Iiyama R. (2003). Regulation of murine inflammatory bowel disease by CD25+ and CD25- CD4+ glucocorticoid-induced TNF receptor family-related gene+ regulatory T cells. *J. Immunol.* 171 708–716 12847237 10.4049/jimmunol.171.2.708

[B215] Valzasina B. Guiducci C. Dislich H. Killeen N. Weinberg A. D. Colombo M. P. (2005). Triggering of OX40 (CD134) on CD4(+)CD25+ T cells blocks their inhibitory activity: a novel regulatory role for OX40 and its comparison with GITR. *Blood* 105 2845–2851 15591118 10.1182/blood-2004-07-2959

[B216] van der Aar A. M. de Groot R. Sanchez-Hernandez M. Taanman E. W. van Lier R. A. Teunissen M. B. (2011). Cutting edge: virus selectively primes human Langerhans cells for CD70 expression promoting CD8+ T cell responses. *J. Immunol.* 187 3488–3492 21880979 10.4049/jimmunol.1101105

[B217] van der Werf N. Redpath S. A. Phythian-Adams A. T. Azuma M. Allen J. E. Maizels R. M. (2011). Th2 responses to helminth parasites can be therapeutically enhanced by, but are not dependent upon, GITR–GITR ligand costimulation in vivo. *J. Immunol.* 187 1411–1420 21705620 10.4049/jimmunol.1100834 PMC3407370

[B218] van Lier R. A. Borst J. Vroom T. M. Klein H. Van Mourik P. Zeijlemaker W. P. (1987). Tissue distribution and biochemical and functional properties of Tp55 (CD27), a novel T cell differentiation antigen. *J. Immunol.* 139 1589–1596 2442250

[B219] Van Nuffel A. M. Benteyn D. Wilgenhof S. Corthals J. Heirman C. Neyns B. (2012). Intravenous and intradermal TriMix-dendritic cell therapy results in a broad T-cell response and durable tumor response in a chemorefractory stage IV-M1c melanoma patient 3. *Cancer Immunol. Immunother.* 61 1033–1043 22159452 10.1007/s00262-011-1176-2 PMC11028719

[B220] Van Seventer G. A. Shimizu Y. Horgan K. J. Shaw S. (1990). The LFA-1 ligand ICAM-1 provides an important costimulatory signal for T cell receptor-mediated activation of resting T cells. *J. Immunol.* 144 4579–4586 1972160

[B221] Vinay D. S. Kwon B. S. (1998). Role of 4-1BB in immune responses. *Semin. Immunol.* 10 481–489 9826581 10.1006/smim.1998.0157

[B222] Vincenti F. Dritselis A. Kirkpatrick P. (2011). Belatacept. *Nat. Rev. Drug Discov.* 10 655–656 21878974 10.1038/nrd3536

[B223] Vlad G. D’Agati V. D. Zhang Q. Y. Liu Z. Ho E. K. Mohanakumar T. (2008). Immunoglobulin-like transcript 3-Fc suppresses T-cell responses to allogeneic human islet transplants in hu-NOD/SCID mice. *Diabetes* 57 1878–1886 18420485 10.2337/db08-0054 PMC2453624

[B224] Vonderheide R. H. Dutcher J. P. Anderson J. E. Eckhardt S. G. Stephans K. F. Razvillas B. (2001). Phase I study of recombinant human CD40 ligand in cancer patients. *J. Clin. Oncol.* 19 3280–3287 11432896 10.1200/JCO.2001.19.13.3280

[B225] Vonderheide R. H. Flaherty K. T. Khalil M. Stumacher M. S. Bajor D. L. Hutnick N. A. (2007). Clinical activity and immune modulation in cancer patients treated with CP-870,893, a novel CD40 agonist monoclonal antibody. *J. Clin. Oncol.* 25 876–883 17327609 10.1200/JCO.2006.08.3311

[B226] Wakkach A. Cottrez F. Groux H. (2001). Differentiation of regulatory T cells 1 is induced by CD2 costimulation. *J. Immunol.* 167 3107–3113 11544295 10.4049/jimmunol.167.6.3107

[B227] Walunas T. L. Bakker C. Y. Bluestone J. A. (1996). CTLA-4 ligation blocks CD28-dependent T cell activation. *J. Exp. Med.* 183 2541–2550 8676075 10.1084/jem.183.6.2541 PMC2192609

[B228] Wang J. Guo Z. Dong Y. Kim O. Hart J. Adams A. (2003). Role of 4-1BB in allograft rejection mediated by CD8+ T cells. *Am. J. Transplant.* 3 543–551 12752310 10.1034/j.1600-6143.2003.00088.x

[B229] Wang S. Zhu G. Chapoval A. I. Dong H. Tamada K. Ni J. (2000). Costimulation of T cells by B7-H2, a B7-like molecule that binds ICOS. *Blood* 96 2808–2813 11023515

[B230] Wang X. Hao J. Metzger D. L. Ao Z. Chen L. Ou D. (2012a). B7-H4 treatment of T cells inhibits ERK, JNK, p38, and AKT activation. *PLoS ONE * 7:e28232 10.1371/journal.pone.0028232 PMC3251556 22238573

[B231] Wang X. Hao J. Metzger D. L. Mui A. Lee I. F. Akhoundsadegh N. (2012b). Endogenous expression of B7-H4 improves long-term murine islet allograft survival. *Transplantation* 95 94–99 23192157 10.1097/TP.0b013e318277229d

[B232] Ware C. F. Sedy J. R. (2011). TNF Superfamily Networks: bidirectional and interference pathways of the herpesvirus entry mediator (TNFSF14). *Curr. Opin. Immunol.* 23 627–631 21920726 10.1016/j.coi.2011.08.008 PMC3222271

[B233] Wassink L. Vieira P. L. Smits H. H. Kingsbury G. A. Coyle A. J. Kapsenberg M. L. (2004). ICOS expression by activated human Th cells is enhanced by IL-12 and IL-23: increased ICOS expression enhances the effector function of both Th1 and Th2 cells. *J. Immunol.* 173 1779–1786 15265908 10.4049/jimmunol.173.3.1779

[B234] Watanabe N. Gavrieli M. Sedy J. R. Yang J. Fallarino F. Loftin S. K. (2003). BTLA is a lymphocyte inhibitory receptor with similarities to CTLA-4 and PD-1. *Nat. Immunol.* 4 670–679 12796776 10.1038/ni944

[B235] Xu Y. Kolber-Simonds D. Hope J. A. Bazin H. Latinne D. Monroy R. (2004). The anti-CD2 monoclonal antibody BTI-322 generates unresponsiveness by activation-associated T cell depletion. *Clin. Exp. Immunol.* 138 476–483 15544625 10.1111/j.1365-2249.2004.02650.x PMC1809246

[B236] Ye Q. Fraser C. C. Gao W. Wang L. Busfield S. J. Wang C. (2002). Modulation of LIGHT–HVEM costimulation prolongs cardiac allograft survival. *J. Exp. Med.* 195 795–800 11901205 10.1084/jem.20012088 PMC2193745

[B237] Ying H. Yang L. Qiao G. Li Z. Zhang L. Yin F. (2010). Cutting edge: CTLA-4–B7 interaction suppresses Th17 cell differentiation. *J. Immunol.* 185 1375–1378 20601598 10.4049/jimmunol.0903369 PMC2915549

[B238] Zang X. Allison J. P. (2007). The B7 family and cancer therapy: costimulation and coinhibition. *Clin. Cancer Res.* 13 5271–5279 17875755 10.1158/1078-0432.CCR-07-1030

[B239] Zhu C. Anderson A. C. Schubart A. Xiong H. Imitola J. Khoury S. J. (2005). The Tim-3 ligand galectin-9 negatively regulates T helper type 1 immunity. *Nat. Immunol.* 6 1245–1252 16286920 10.1038/ni1271

[B240] Zhu G. Augustine M. M. Azuma T. Luo L. Yao S. Anand S. (2009). B7-H4-deficient mice display augmented neutrophil-mediated innate immunity. *Blood* 113 1759–1767 19109567 10.1182/blood-2008-01-133223 PMC2647680

[B241] Zitvogel L. Kroemer G. (2012). Targeting PD-1/PD-L1 interactions for cancer immunotherapy. *Oncoimmunology* 1 1223–1225 23243584 10.4161/onci.21335 PMC3518493

[B242] Zucchelli S. Holler P. Yamagata T. Roy M. Benoist C. Mathis D. (2005). Defective central tolerance induction in NOD mice: genomics and genetics. *Immunity* 22 385–396 15780994 10.1016/j.immuni.2005.01.015

